# A Computational Model of Loss of Dopaminergic Cells in Parkinson's Disease Due to Glutamate-Induced Excitotoxicity

**DOI:** 10.3389/fncir.2019.00011

**Published:** 2019-02-25

**Authors:** Vignayanandam Ravindernath Muddapu, Alekhya Mandali, V. Srinivasa Chakravarthy, Srikanth Ramaswamy

**Affiliations:** ^1^Computational Neuroscience Lab, Department of Biotechnology, Bhupat and Jyoti Mehta School of Biosciences, IIT-Madras, Chennai, India; ^2^Department of Psychiatry, Behavioural and Clinical Neuroscience Institute, University of Cambridge, Cambridge, United Kingdom; ^3^Blue Brain Project, Brain and Mind Institute, EPFL, Geneva, Switzerland

**Keywords:** Parkinson's disease, excitotoxicity, deep brain stimulation, Izhikevich neuron model, Substantia Nigra pars compacta, SubThalamic Nucleus, Globus Pallidus externa, metabolic disorders

## Abstract

Parkinson's disease (PD) is a neurodegenerative disease associated with progressive and inexorable loss of dopaminergic cells in Substantia Nigra pars compacta (SNc). Although many mechanisms have been suggested, a decisive root cause of this cell loss is unknown. A couple of the proposed mechanisms, however, show potential for the development of a novel line of PD therapeutics. One of these mechanisms is the peculiar metabolic vulnerability of SNc cells compared to other dopaminergic clusters; the other is the SubThalamic Nucleus (STN)-induced excitotoxicity in SNc. To investigate the latter hypothesis computationally, we developed a spiking neuron network-model of SNc-STN-GPe system. In the model, prolonged stimulation of SNc cells by an overactive STN leads to an increase in ‘stress' variable; when the stress in a SNc neuron exceeds a stress threshold, the neuron dies. The model shows that the interaction between SNc and STN involves a positive-feedback due to which, an initial loss of SNc cells that crosses a threshold causes a runaway-effect, leading to an inexorable loss of SNc cells, strongly resembling the process of neurodegeneration. The model further suggests a link between the two aforementioned mechanisms of SNc cell loss. Our simulation results show that the excitotoxic cause of SNc cell loss might initiate by weak-excitotoxicity mediated by energy deficit, followed by strong-excitotoxicity, mediated by a disinhibited STN. A variety of conventional therapies were simulated to test their efficacy in slowing down SNc cell loss. Among them, glutamate inhibition, dopamine restoration, subthalamotomy and deep brain stimulation showed superior neuroprotective-effects in the proposed model.

## 1. Introduction

There is a long tradition of investigation into the etiology and pathogenesis of Parkinson's Disease (PD) that seeks to link molecular (pesticides, oxidative stress, protein dysfunction etc.) (Hwang, 2013; Ortiz et al., 2016; Chiti and Dobson, 2017; Anselmi et al., 2018; Stykel et al., 2018) and subcellular (mitochondrial dysfunction etc.) (Henchcliffe and Beal, 2008; Reeve et al., 2018; Tsai et al., 2018) factors with the disease development. However, recent years see the emergence of two novel lines of investigation into PD pathogenesis. These approaches, that aim to understand the PD pathology at the cellular and network level, mark a significant deviation from the traditional approaches (Rodriguez et al., 1998; Pissadaki and Bolam, 2013; Pacelli et al., 2015; Chakravarthy and Moustafa, 2018).

The first approach believes the primary factor that causes the degeneration of dopaminergic neurons of Substantia Nigra pars compacta (SNc) is its high metabolic requirements. SNc neurons are one of the most vulnerable and energy consuming neuronal clusters, due to their structural and functional properties. Here, we have listed down some of the plausible factors which make SNc cells to be most susceptible.

- *Complex axonal arbors*: Large axonal arborisation which requires large amounts of energy to drive currents along these axons (Bolam and Pissadaki, 2012; Pissadaki and Bolam, 2013).
- *Reactive neurotransmitter*: When a reactive neurotransmitter like dopamine is present in excess, it would readily oxidizes with proteins, nucleic acids and lipids (Sulzer, 2007) eventually leading to neurodegeneration. One of the mechanisms for sequestration of excess cytosolic dopamine is packing of dopamine into synaptic vesicles through vesicular monoamine transporter-2 (VMAT-2) using H+ concentration gradient which is maintained by H+-ATPase. In addition to that, in the case of substantia nigra, the expression of VMAT2 is lower than in the ventral tegmental area (Liang et al., 2004; Mosharov et al., 2009) which likely causes dopamine-mediated oxidative stress in SNc cells.
- *Auto-rhythmicity*: Uses L-type calcium channels for maintaining the pace-making type of firing which in turn requires higher amounts of energy to maintain calcium homeostasis (Surmeier et al., 2017) and lower expression of calcium-binding proteins (lower capacity of calcium buffering mechanism) adds additional burden on the cell's metabolic activity (German et al., 1992).
- *NMDA synaptic activation*: Due to pacemaker type of firing, magnesium blockage of NMDA receptors is ineffective, resulting in substantial NMDA receptor currents even with weak glutamatergic inputs resulting in additional burden to maintain calcium homeostasis; the resulting energy deficiency leads to excitotoxicity (Rodriguez et al., 1998; Surmeier et al., 2010).
- *Prone to neuroinflammation*: Astrocytes play a modulatory role in microglial activation (McGeer and McGeer, 2008; Glass et al., 2010; Rocha et al., 2012) and any miscommunication between them results in neuroinflammation which eventually leads to neurodegeneration (Waak et al., 2009; Booth et al., 2017). The risk of inflammation in SNc neurons is high due to the small proportion of astrocytes regulating the huge population of microglia in this region (Lawson et al., 1990; Whitton, 2007; Mena and García de Yébenes, 2008). It has been reported that neuromelanin can induce microglial activation (Zecca et al., 2008; Zhang et al., 2011). SNc neurons are more susceptible to neuro-melanin induced inflammation compared to VTA neurons due to their high neuro-melanin biosynthesis as a result of underexpression of VMAT2 (Peter et al., 1995; Liang et al., 2004).
- *Weak microvasculature*: SNc neurons are more prone to environmental toxins due to weak surrounding cerebral microvasculature (Rite et al., 2007).

Since the metabolic demands of SNc neurons are particularly high when compared to any other neuronal types (Sulzer, 2007) including neurons of other dopaminergic systems (Bolam and Pissadaki, 2012; Pacelli et al., 2015; Giguère et al., 2018), any sustained insufficiency in the supply of energy can result in cellular degeneration, characteristic of PD (Mergenthaler et al., 2013).

According to the second approach, the overactivity of SubThlamic Nucleus (STN) in PD causes excessive release of glutamate to the SNc, which in turns causes degeneration of SNc neurons by glutamate excitotoxicity (Rodriguez et al., 1998). The above two approaches are interrelated and not entirely independent as one form of excitotoxicity - the ‘weak excitotoxicity' - is thought to have its roots in impaired cellular metabolism (Albin and Greenamyre, 1992). Therefore, the insight behind these new lines of investigation is the mismatch in energy supply and demand which could be a primary factor underlying neurodegeneration in PD. Such a mismatch is more likely to take place in special nuclei like SNc due to their peculiar metabolic vulnerability (Bolam and Pissadaki, 2012; Pissadaki and Bolam, 2013; Sulzer and Surmeier, 2013; Pacelli et al., 2015; Surmeier et al., 2017; Giguère et al., 2018). Similar ideas have been proffered to account for other forms of neurodegenerative diseases such as Huntington's disease, Alzheimer's disease, and amyotrophic lateral sclerosis (Beal et al., 1993; Johri and Beal, 2012; Gao et al., 2017).

If metabolic factors are indeed the underlying reason behind PD pathogenesis, it is a hypothesis that deserves closer attention and merits a substantial investment of time and effort for an in-depth study. This is because any positive proof regarding the role of metabolic factors puts an entirely new spin on PD research. Several researchers proposed that systems-level energy imbalance probably a principal cause of PD (Wellstead and Cloutier, 2011; Bolam and Pissadaki, 2012; Pacelli et al., 2015). Unlike current therapeutic approaches that manage the symptoms rather than provide a cure, the new approach can in principle point to a more lasting solution. If inefficient energy delivery or energy transformation mechanisms are the reason behind degenerative cell death, relieving the metabolic load on the vulnerable neuronal clusters, by intervening through current clinically approved therapeutics (such as brain stimulation and pharmacology) could prove to be effective treatments (Adhihetty and Beal, 2008; Spieles-Engemann et al., 2010; Seidl and Potashkin, 2011; Musacchio et al., 2017).

In this paper, with the help of computational models, we investigate the hypothesis that the cellular energy deficiency in SNc could be the primary cause of SNc cell loss in PD. *The higher metabolic demand of SNc cells due to their unique molecular characteristics, complex morphologies, and other energy-demanding features perhaps make them more vulnerable to energy deficit*. Therefore, prolonged energy deprivation or insufficiency in such cells creates metabolic stress, eventually leading to neurodegeneration. If we can aim to reduce the metabolic stress on SNc cells, we can delay the progression of cell loss in PD.

In the proposed modeling study, we focus on excitotoxicity in SNc caused by STN which is precipitated by energy deficiency (Greene and Greenamyre, 1996) and exploring simulated therapeutic strategies for slowing down SNc cell loss. With the help of computational models of neurovascular coupling, our group had earlier explored the effect of rhythms of energy delivery from the cerebrovascular system on neural function (Gandrakota et al., 2010; Chander and Chakravarthy, 2012; Chhabria and Chakravarthy, 2016; Philips et al., 2016). Recently, we proposed a preliminary computational spiking network model of STN-mediated excitotoxicity in SNc with a slightly abstract treatment of apoptosis (Muddapu and Chakravarthy, 2017). Building on the previous version of the model, we have improved the excitotoxicity model by incorporating more biologically plausible dopamine plasticity and also explored the therapeutic strategies to slow down or halt the SNc cell loss.

## 2. Materials and Methods

**Figure 1:**
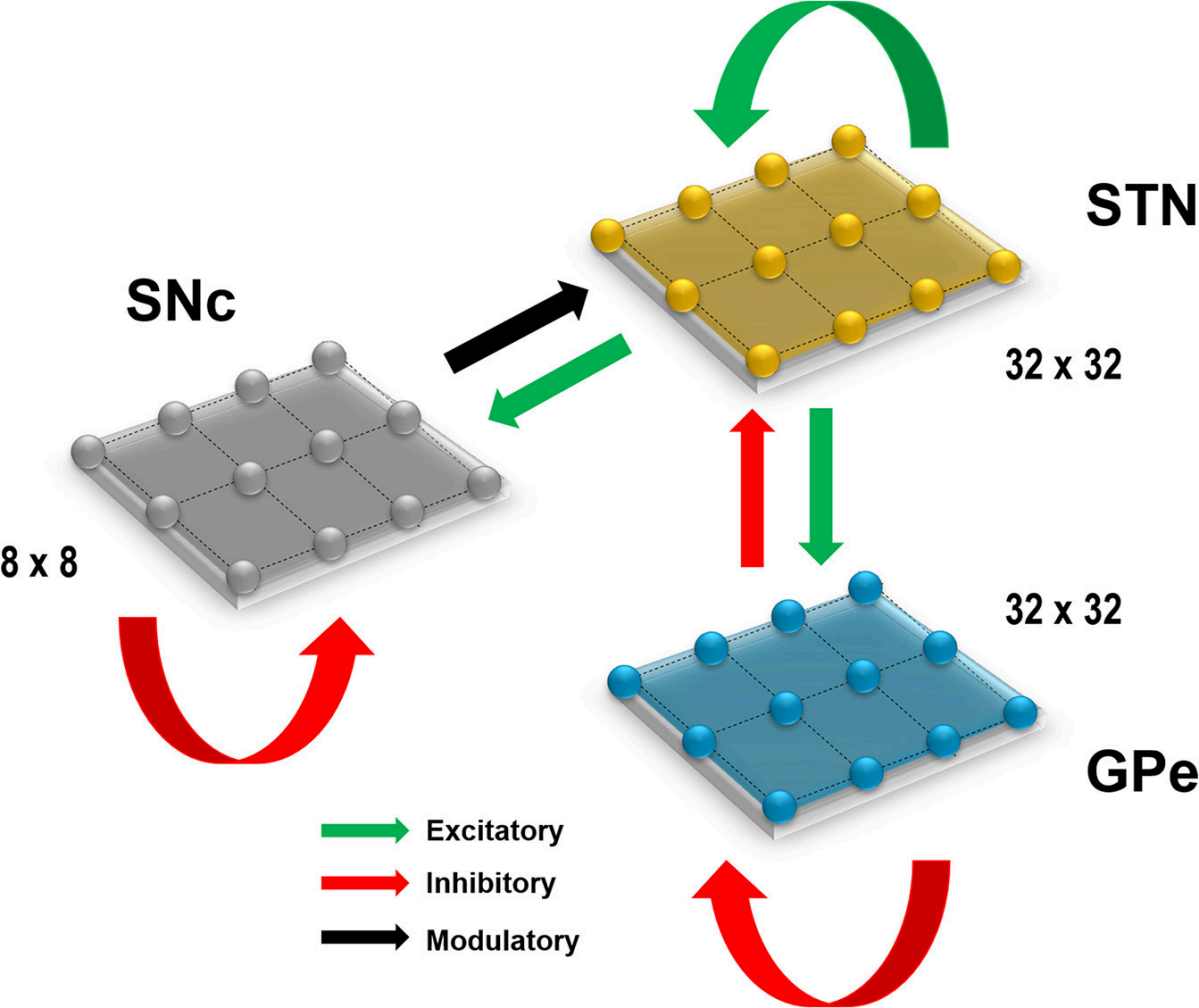
Proposed model architecture. The model architecture of the proposed model of STN-mediated excitotoxicity in SNc. STN, SubThalamic Nucleus; SNc, Substantia Nigra pars compacta; GPe, Globus Pallidus externa.

All the nuclei were modeled as Izhikevich 2D neurons (Figure 1). All the simulations were performed by numerical integration using MATLAB (RRID:SCR_001622) with a time step (dt) of 0.1 s. The average time for 50-s simulation was around 10 h, and it reduced to 5 h when ran on GPU card (Nvidia Quadro K620).

### 2.1. Izhikevich Neuron Model

Computational neuroscientists are often required to select the level at which a given model of interest must cast, i.e., biophysical-level, conductance-based modeling level, spiking neuron-level or rate-coded level. Biophysical models capture a more biologically detailed dynamics but are computationally expensive whereas rate-coded, point-neuron models are computationally inexpensive but possess less biologically detailed dynamics. To overcome this predicament, Izhikevich (2003) developed spiking neuron models that are comparatively computationally inexpensive yet capture various neuronal dynamics. The proposed model of excitotoxicity consists of SNc, STN, and Globus Pallidus externa (GPe) modeled using Izhikevich neuron models arranged in a 2D lattice (Figure 1). The population sizes of these nuclei in the model were selected based on the neuronal numbers of these nuclei in rat basal ganglia (Oorschot, 1996). The Izhikevich parameters for STN and GPe were adapted from Michmizos and Nikita (2011) and Mandali et al. (2015) and the parameters for SNc were adapted from Cullen and Wong-Lin (2015). The firing rates of these neuronal types were tuned to match the published data (Modolo et al., 2007; Tripathy et al., 2014) by varying the external bias current $\left(I_{ij}^{x}\right)$. All parameters values are given in the Table 1. The Izhikevich model consists of two variables, one for membrane potential (*v*^*x*^) and the other one for membrane recovery variable (*u*^*x*^).

(1) $$\begin{array}{r} \frac{dv_{ij}^{x}}{dt}=0.04{\left(v_{ij}^{x}\right)}^{2}+5v_{ij}^{x}+140-u_{ij}^{x}+I_{ij}^{x}+I_{ij}^{syn} \end{array}$$

(2) $$\begin{array}{r} \frac{du_{ij}^{x}}{dt}=a\left(bv_{ij}^{x}-u_{ij}^{x}\right) \end{array}$$

(3) $$if\;v_{ij}^{x}\geq v_{peak}\;\left\{\;\begin{array}{c} v_{ij}^{x}\leftarrow c\\ u_{ij}^{x}\leftarrow u_{ij}^{x}+d \end{array}\;\right\}$$

where, $v_{ij}^{x}$, $u_{ij}^{x}$, $I_{ij}^{syn}$, and $I_{ij}^{x}$ are the membrane potential, the membrane recovery variable, the total amount synaptic current received and the external current applied to neuron *x* at location (*i, j*), respectively, *v*_*peak*_ is the maximum membrane voltage set to neuron (+30 mV) with *x* being GPe or SNc or STN neuron.

**Table 1:** Parameter values used in the proposed model.

**Parameter(s)**	**STN**	**SNc**	**GPe**
Izhikevich parameters (a, b, c, d)	a = 0.005, b = 0.265, c = −65, d = 1.5	a = 0.0025, b = 0.2, c = −55, d = 2	a = 0.1, b = 0.2, c = −65, d = 2
External current (*I*^*x*^)	*I*^*STN*^= 3	*I*^*SNc*^= 9	*I*^*GPe*^= 4.25
Number of laterals (*nlat*_*x*_)	*nlat*_*STN*_ = 11	*nlat*_*SNc*_ = 5	*nlat*_*GPe*_ = 15
Radius of Gaussian laterals (*R*_*x*_)	*R*_*STN*_ = 1.4	*R*_*SNc*_ = 1.6	*R*_*GPe*_ = 1.6
Synaptic strength within laterals (*A*_*x*_)	*A*_*STN*_ = 1.3	*A*_*SNc*_ = 0.1	*A*_*GPe*_ = 0.1
Time decay constant for AMPA (τ_*AMPA*_)	6 ms	6 ms	6 ms
Time decay constant for NMDA (τ_*NMDA*_)	160 ms	160 ms	160 ms
Time decay constant for GABA (τ_*GABA*_)	4 ms	4 ms	4 ms
Synaptic potential of AMPA receptor (*E*_*AMPA*_)	0 mV	0 mV	0 mV
Synaptic potential of NMDA receptor (*E*_*NMDA*_)	0 mV	0 mV	0 mV
Synaptic potential of GABA receptor (*E*_*GABA*_)	−60 mV	−60 mV	−60 mV
Effect on the post-synaptic current (*cd*2)	0.1	0.1	0.1
Concentration of Magnesium (*Mg*^2+^)	1 nM	1 nM	1 nM

*ms, milliseconds; mV, millivolts; nM, nanomolar*.

### 2.2. Synaptic Connections

The presence of excitatory synaptic connectivity from STN to SNc was observed from anatomical and electrophysiology studies (Kita and Kitai, 1987; Smith and Grace, 1992; Hamani et al., 2004, 2017) and these connections might take part in controlling the bursting activity of SNc (Smith and Grace 1992). The sizes (number of neurons) of SNc (8 × 8), STN (32 × 32) and GPe (32 × 32) nuclei in the model were selected such that they match the proportions as observed in the rat basal ganglia (Oorschot, 1996). We also modeled convergent projections from STN to SNc as per anatomical observations (Oorschot, 1996). Similarly, the synaptic connectivity between GPe and STN was considered one-to-one as in Dovzhenok and Rubchinsky (2012) and Mandali et al. (2015). The equations used to model synaptic connectivity are

(4) $$\begin{array}{r} \tau_{Recep}*\frac{dh_{ij}^{x\to y}}{dt}=-h_{ij}^{x\to y}+S_{ij}^{x}\left(t\right) \end{array}$$

(5) $$\begin{array}{r} I_{ij}^{x\to y}\left(t\right)=W_{x\to y}*h_{ij}^{x\to y}\left(t\right)*\left(E_{Recep}-V_{ij}^{y}\left(t\right)\right) \end{array}$$

The NMDA currents are regulated by voltage-dependent magnesium channel (Jahr and Stevens, 1990) which was modeled as,

(6) $$\begin{array}{r} B_{ij}\left(v_{ij}\right)=\frac{1}{1\;+\;\left(\frac{Mg^{2+}}{3.57}*e^{-0.062*V_{ij}^{y}\left(t\right)}\right)} \end{array}$$

where, $S_{ij}^{x}$ is the spiking activity of neuron *x* at time *t*, τ_*Recep*_ is the decay constant for synaptic receptor, $h_{ij}^{x\to y}$ is the gating variable for the synaptic current from *x* to *y*, *W*_*x*→*y*_ is the synaptic weight from neuron *x* to *y*, *Mg*^2+^ is the magnesium ion concentration, $V_{ij}^{y}$ is the membrane potential of the neuron *y* for the neuron at the location (*i, j*) and *E*_*Recep*_ is the receptor associated synaptic potential (Recep = NMDA/AMPA/GABA). The time constants of NMDA, AMPA, and GABA in GPe, SNc, and STN were chosen from Götz et al. (1997) are given in the Table 1.

### 2.3. Lateral Connections

Lateral connections are similar to collaterals of a neuron, and here it is defined as connections within each neuronal population. Earlier studies show the presence of lateral connections in STN (Kita et al., 1983) and GPe (Kita and Kita, 1994). In the case of SNc, the GABAergic interneurons were observed and their control of SNc activity revealed by immunohistochemistry studies (Hebb and Robertson, 1999; Tepper and Lee, 2007). To simplify the model, the GABAergic interneurons were replaced by GABAergic lateral connections in SNc population. Experimental studies show that synaptic current from lateral connections follows Gaussian distribution (Lukasiewicz and Werblin, 1990) that is, nearby neurons will have more influence than distant neurons. The lateral connections in various modules in the current network (STN, GPe, and SNc) were modeled as Gaussian neighborhoods (Mandali et al., 2015) and the parameters used are given in the Table 1. Each neuron receives synaptic input from a set number of neighboring neurons located in a 2D grid of size nxn.

(7) $$\begin{array}{r} w_{ij,pq}^{m\to m}=A_{m}*e^{\frac{-d_{ij,pq}^{2}}{R_{m}^{2}}} \end{array}$$

(8) $$\begin{array}{r} d_{ij,pq}^{2}={\left(i-p\right)}^{2}+{\left(j-q\right)}^{2} \end{array}$$

where, $w_{ij,pq}^{m\to m}$ is the lateral connection weight of neuron type m at location (*i, j*), $d_{ij,pq}^{2}$ is the distance from center neuron (*p, q*), *R*_*m*_ is the variance of Gaussian, *A*_*m*_ is the strength of lateral synapse, m = GPe or SNc or STN.

### 2.4. Effect of DA on Synaptic Plasticity

Several experimental studies demonstrate dopamine-dependent synaptic plasticity in STN (Hassani et al., 1997; Magill et al., 2001; Yang et al., 2016) and GPe (Magill et al., 2001; Mamad et al., 2015). Experimental observations show an increase in synchrony in STN (Bergman et al., 1994, 1998) and GPe populations (Bergman et al., 1998) at low DA levels. The effect of low DA was implemented in the model by increasing in lateral connections strength in STN population as in Hansel et al. (1995) and similarly decrease in lateral connections strength in GPe as in Wang and Rinzel (1993). Similarly, SNc populations also showed an increase in synchrony at low DA levels (Hebb and Robertson, 1999; Vandecasteele et al., 2005; Tepper and Lee, 2007; Ford, 2014) which was modeled similarly to the model of DA-modulated GPe.

We modeled DA effect on the network as follows: as DA level increases, the strength of the lateral connections in STN decreases whereas, in GPe and SNc, lateral connection weights become stronger. As the lateral connection weights directly controls the amount of synaptic current each neuron receives. All the neurons in STN population will tend to fire together as the lateral connection weights increases (due to excitatory synapses). However, in the case of SNc and GPe it is contrary, that is, all the neurons will not tend to fire together as the lateral connection weights increases (due to inhibitory synapses). Lateral strength was modulated by DA as follows,

(9) $$\begin{array}{r} A_{STN}=s_{STN}^{max}*e^{\left(-cd_{stn}*DA_{s}\left(t\right)\right)} \end{array}$$

(10) $$\begin{array}{r} A_{GPe}=s_{GPe}^{max}*e^{\left(cd_{gpe}*DA_{s}\left(t\right)\right)} \end{array}$$

(11) $$\begin{array}{r} A_{SNc}=s_{SNc}^{max}*e^{\left(cd_{snc}*DA_{s}\left(t\right)\right)} \end{array}$$

where, $s_{STN}^{max}$, $s_{GPe}^{max}$, and $s_{SNc}^{max}$ are strength of the lateral connections at the basal spontaneous activity of the population without any external influence in STN, GPe, and SNc, respectively. *cd*_*stn*_, *cd*_*gpe*_, and *cd*_*snc*_ were the factors by which dopamine affects the lateral connections in STN, GPe, and SNc populations, respectively, *DA*_*s*_(*t*) is the instantaneous dopamine level which is the spatial average activity of all the neurons in SNc.

According to experimental studies, DA causes post-synaptic effects on afferent currents in GPe and STN (Shen and Johnson, 2000; Smith and Kieval, 2000; Magill et al., 2001; Cragg et al., 2004; Fan et al., 2012). DA causes post-synaptic effects on afferent currents in SNc through somatodendritic DA receptors (Jang et al., 2011; Courtney et al., 2012; Ford, 2014). Thus, we included a factor (*cd*2), which regulates the effect of DA on synaptic currents of GPe, SNc, and STN. As observed in Kreiss et al. (1997), as DA level increases, the regulated current decreases as follows:

(12) $$\begin{array}{r} W_{x\to y}=\left(1-cd2*DA_{s}\left(t\right)\right)*w_{x\to y} \end{array}$$

where, *W*_*x*→*y*_ is the synaptic weight (*STN* → *GPe*, *GPe* → *STN*, *STN* → *STN*, *GPe* → *GPe*, *STN* → *SNc*, *SNc* → *SNc*), (*cd*2) is the parameter that affects the post-synaptic current, *DA*_*s*_(*t*) is the instantaneous dopamine level which is the spatial average activity of all the neurons in SNc.

### 2.5. Total Synaptic Current Received by Each Neuron

***STN:***

The total synaptic current received by a STN neuron at lattice position (*i, j*) is the summation of lateral glutamatergic input from other STN neurons considering both NMDA and AMPA currents and the GABAergic input from the GPe neurons.

(13) $$\begin{array}{r} I_{ij}^{STNsyn}=I_{ij}^{NMDAlat}+I_{ij}^{AMPAlat}+I_{ij}^{GABA\to STN} \end{array}$$

where, $I_{ij}^{NMDAlat}$ and $I_{ij}^{AMPAlat}$ are the lateral glutamatergic current from other STN neurons considering both NMDA and AMPA receptors, respectively, $I_{ij}^{GABA\to STN}$ is the GABAergic current from GPe neuron.

***GPe:***

The total synaptic current received by a GPe neuron at lattice position (*i, j*) is the summation of the lateral GABAergic current from other GPe neurons and the glutamatergic input from the STN neurons considering both NMDA and AMPA currents.

(14) $$\begin{array}{r} I_{ij}^{GPesyn}=I_{ij}^{GABAlat}+I_{ij}^{NMDA\to GPe}+I_{ij}^{AMPA\to GPe} \end{array}$$

where, $I_{ij}^{GABAlat}$ is the lateral GABAergic current from other GPe neurons, $I_{ij}^{NMDA\to GPe}$ and $I_{ij}^{AMPA\to GPe}$ are the glutamatergic current from STN neuron considering both NMDA and AMPA receptors, respectively.

***SNc:***

The total synaptic current received by a SNc neuron at lattice position (*i, j*) is the summation of the lateral GABAergic current from other SNc neurons and the glutamatergic input from the STN neurons considering both NMDA and AMPA currents.

(15) $$\begin{array}{r} I_{ij}^{SNcsyn}=I_{ij}^{GABAlat}+I_{ij}^{NMDA\to SNc}+I_{ij}^{AMPA\to SNc} \end{array}$$

where, $I_{ij}^{GABAlat}$ is the lateral GABAergic current from other SNc neurons, $I_{ij}^{NMDA\to SNc}$ and $I_{ij}^{AMPA\to SNc}$ are the glutamatergic current from STN neuron considering both NMDA and AMPA receptors, respectively.

### 2.6. Neurodegeneration

According to Rodriguez et al. (1998), dopamine deficiency in SNc leads to disinhibition and overactivity of the STN, which in turn causes excitotoxic damage to its target structures, including SNc itself. In order to simulate the SNc excitotoxicity induced by STN, we incorporate a mechanism of programmed cell death, whereby an SNc cell under high stress kills itself. The stress on a given SNc cell was calculated based on the firing history of the cell - higher firing activity causes higher stress.

The stress of each SNc neuron at lattice position (*i, j*) at time *t* due to excess firing is calculated as,

(16) $$\begin{array}{r} \tau_{stress}*\frac{dQ_{ij}^{x}}{dt}=-Q_{ij}^{x}+r_{ij}^{x}\left(t\right) \end{array}$$

where, $r_{ij}^{x}\left(t\right)$ is instantaneous mean firing rate of a SNc neuron at lattice position (*i, j*) at time *t*, calculated with a fixed sliding window Δ*t* (1 s) (Dayan and Abbott, 2005) as,

(17) $$\begin{array}{r} r_{ij}^{x}\left(t\right)=\frac{1}{\Delta t}\int_{t-\Delta t}^{t}d\tau \rho \left(\tau \right) \end{array}$$

and,

(18) $$\begin{array}{r} \rho \left(\tau \right)=\overset{n}{\underset{i=1}{\sum }}\delta \left(t-t_{i}\right)\\ \text{Sequence of spike timing: }t_{i}=1,2,3\ldots n \end{array}$$

If stress variable $\left(Q_{ij}^{x}\right)$ of a SNc neuron at lattice position (*i, j*) crosses certain threshold (*S*_*thres*_) then that particular SNc neuron will be eliminated (Iglesias and Villa, 2008).

(19) $$\begin{array}{rclllll} if & \; & Q_{ij}^{x}\left(t\right)>S_{thres}, & \; & then & \; & v_{ij}^{x}\left(t\right)=0 \end{array}$$

#### 2.6.1. Estimating Rate of Degeneration

For a given course of SNc cell loss, the half-life is the time taken for half of the SNc cells to be lost (*t*_1/2_). The following equation was used to estimate the number of SNc cells (*N*_*sc*_(*t*)) for a given course that survived after a given time *t*.

(20) $$\begin{array}{r} N_{sc}\left(t\right)=N_{sc}^{0}*e^{-\lambda t} \end{array}$$

where, λ is the rate of degeneration (*sec*^−1^), $N_{sc}^{0}$ is the number of surviving SNc cells at *t* = 0.

To estimate the rate of degeneration λ from a given course of SNc cell loss, the following equation was used,

(21) $$\begin{array}{r} \lambda =\frac{\ln2}{t_{1/2}} \end{array}$$

The instantaneous rate of degeneration λ(*t*) was calculated by the following equation,

(22) $$\begin{array}{r} \lambda \left(t\right)=\frac{\ln\left(N_{sc}\left(t\right)\right)\;-\;ln\left(N_{sc}\left(t\;-\;1\right)\right)}{t\;-\;\left(t\;-\;1\right)} \end{array}$$

### 2.7. Neuroprotective Strategies

Pharmacological or surgical therapies that abolish the pathological oscillations in STN or block the receptors on SNc can be neuroprotective and might slow down the progression of SNc cell loss (Rodriguez et al., 1998).

#### 2.7.1. Glutamate Inhibition Therapy

Glutamate drug therapy can have neuroprotective effect on SNc in two ways (1) Inactivation of NMDA (N-methyl-D-aspartate), AMPA (2-amino-3-(5-methyl-3-oxo-1,2-oxazole-4-yl) propanoic acid) or excitatory metabotropic glutamate (Group-I - mGluR1/5) receptors (mGluR) by glutamate antagonists, and (2) Activation of metabotropic glutamate (Group-II/III - mGluR2,3/4,6,7,8) receptors by glutamate agonists. NMDA antagonist MK-801 showed reduction of SNc cell loss in the neurotoxic rats (Turski et al., 1991; Zuddas et al., 1992b; Brouillet and Beal, 1993; Blandini, 2001; Armentero et al., 2006) and primates (Zuddas et al., 1992a,b). AMPA antagonists such as NBQX (Merino et al., 1999), LY-503430 (Murray et al., 2003) and LY-404187 (O'Neill et al., 2004) exhibited neuroprotection of SNc cells in the neurotoxic animal models. mGluR-5 antagonist MPEP and MTEP showed neuroprotection in 6-OHDA lesioned rats (Armentero et al., 2006; Hsieh et al., 2012; Ferrigno et al., 2015; Fuzzati-Armentero et al., 2015) and MPTP-treated primates (Masilamoni et al., 2011), respectively. Broad-spectrum group II (Murray et al., 2002; Battaglia et al., 2003; Vernon et al., 2005) and group III (Vernon et al., 2005; Austin et al., 2010) agonists showed neuroprotection in neurotoxic rats. Selective mGluR2/3 agonist 2R,4R APDC (Chan et al., 2010) and mGluR4 agonist VU0155041 (Betts et al., 2012) significantly attenuated SNc cell loss in 6-OHDA lesioned rats.

The glutamate drug therapy was implemented in the proposed excitotoxicity model by the following criterion,

(23) $$W_{STN\to SNc}(N_{sc},t)=\{\;\begin{array}{cc} \;W_{STN\to SNc}^{0}, & N_{sc}(t)>N_{i}\\ W_{STN\to SNc}^{0}*\delta_{GI}, & N_{sc}(t)\leq N_{i} \end{array}$$

where, *W*_*STN*→*SNc*_(*N*_*sc*_, *t*) is the instantaneous change in synaptic weight of STN to SNc based on the number of surviving SNc neurons at time *t* *N*_*sc*_(*t*) is the instantaneous number of surviving SNc neurons, $W_{STN\to SNc}^{0}$ is the basal connection strength of STN to SNc, δ_*GI*_ is the proportion of glutamate inhibition, *N*_*i*_ is the number representing SNc cell loss 〈*i* = 25% | 50% | 75%〉 at which therapeutic intervention was employed. In the present study, we have considered 25% (cells lost = 16), 50% (cells lost = 32) and 75% (cells lost = 48) SNc cell loss as early, intermediate and late stages of disease progression, respectively.

#### 2.7.2. Dopamine Restoration Therapy

The neuroprotective effects of DA agonists therapy are thought to be due to one or more of the following mechanisms: (1) L-DOPA sparing, (2) Autoreceptor effects, (3) Antioxidant effects, (4) Antiapoptotic effects, and (5) Amelioration of STN-mediated excitotoxicity (Olanow et al., 1998; Grandas, 2000; Schapira, 2003; Zhang and Tan, 2016). In the present study, we focus on the amelioration of STN-mediated excitotoxicity. DA agonists can restore the dopaminergic tone in the dopamine-denervated brain, which results in increased inhibition in STN, thereby diminishing STN-induced excitotoxicity on SNc neurons (Olanow et al., 1998; Schapira and Olanow, 2003; Piccini and Pavese, 2006; Vaarmann et al., 2013).

The dopamine agonist therapy was implemented in the proposed excitotoxicity model by the following criterion,

(24) $$DA(N_{sc},t)=\{\;\begin{array}{cc} \;DA_{s}(t), & \;N_{sc}(t)>N_{i}\\ DA_{s}(t)+\delta_{DAA}, & N_{sc}(t)\leq N_{i} \end{array}$$

where, *DA*(*N*_*sc*_, *t*) is the instantaneous change in dopamine level based on the number of surviving SNc neurons at time *t* *N*_*sc*_(*t*) is the instantaneous number of surviving SNc neurons, *DA*_*s*_(*t*) is the instantaneous dopamine signal from the SNc neurons, δ_*DAA*_ is the proportion of dopamine content restoration, *N*_*i*_ is the number representing SNc cell loss at which therapeutic intervention was employed.

#### 2.7.3. Subthalamotomy

Subthalamotomy is still quite a common treatment amongst patients in advanced stages of PD where patients stop responding to L-DOPA (wearing-off) or chronic L-DOPA therapy results in motor complications such as L-DOPA Induced Dyskinesias (LID) (Alvarez et al., 2009; Obeso et al., 2017). It was reported that STN lesioning exhibits neuroprotective effect which acts as an antiglutamatergic effect in neurotoxic animal models (Piallat et al., 1996; Chen et al., 2000; Carvalho and Nikkhah, 2001; Paul et al., 2004; Wallace et al., 2007; Jourdain et al., 2014).

STN ablation was implemented in the proposed excitotoxicity model by the following criterion,

(25) $$\begin{array}{rclllllll} if & \; & N_{sc}\left(t\right)\leq N_{i}, & \; & then & \; & v_{ij}^{STN} & \; & \left(P_{les},t\right)=0 \end{array}$$

where, *P*_*les*_ is the lesion percentage of STN which is selected from the following range: [5, 10, 20, 40, 60, 80, 100], *N*_*sc*_(*t*) is the instantaneous number of surviving SNc neurons, *N*_*i*_ is the number representing SNc cell loss at which therapeutic intervention was employed.

#### 2.7.4. Deep Brain Stimulation (DBS) in STN

DBS therapy is preferred over ablation therapy of STN due to the potentially irreversible damage to the stimulated brain region in ablation therapy. It has been reported that long-term stimulation (DBS) of STN results in the slowdown of the progression of SNc cell loss in animal models (Benazzouz et al., 2000; Maesawa et al., 2004; Temel et al., 2006; Wallace et al., 2007; Spieles-Engemann et al., 2010; Musacchio et al., 2017).

**Figure 2:**
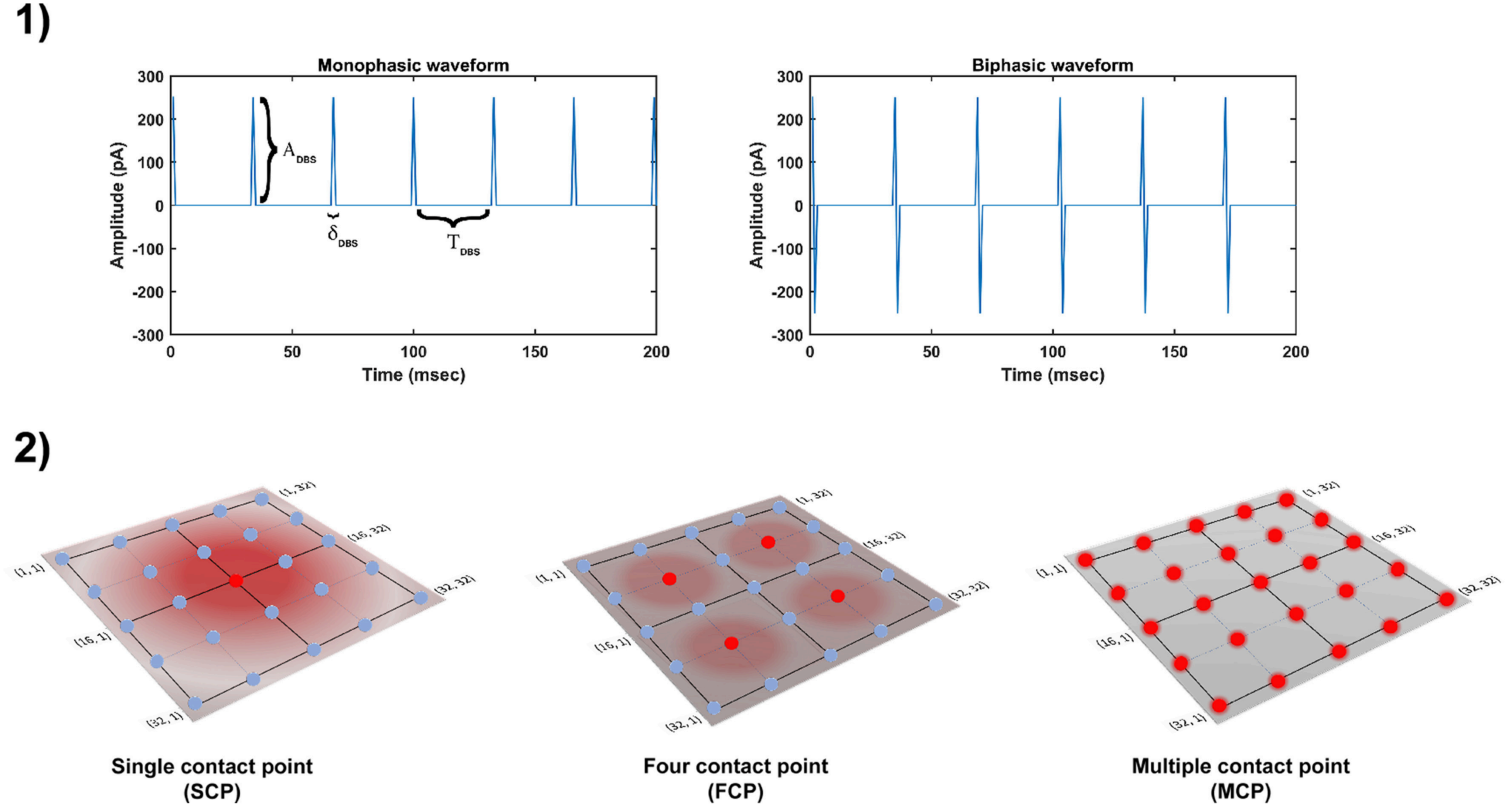
Different DBS protocol used in the study. **(1)** DBS stimulation waveforms. **(2)** DBS stimulation configurations. DBS, Deep Brain Stimulation; *A*_*DBS*_, Amplitude of DBS current pulse; δ_*DBS*_, Pulse width of DBS current pulse; *T*_*DBS*_(1/*f*_*DBS*_), frequency of DBS current pulse; SCP, Single Contact Point; FCP, Four Contact Point; MCP, Multiple Contact Point.

The DBS electrical stimulation was given in the form of current or voltage pulses to the target neuronal tissue (Cogan, 2008). The effect of DBS therapy was modeled as external stimulation current given to the entire or part of the STN module in the form of Gaussian distribution (Rubin and Terman, 2004; Hauptmann and Tass, 2007; Foutz and McIntyre, 2010; Mandali and Chakravarthy, 2016). The DBS parameters such as amplitude (*A*_*DBS*_), frequency $\left(f_{DBS}=\frac{1}{T_{DBS}}\right)$ and pulse width (δ_*DBS*_) were adjusted by using clinical settings as a constraint (Moro et al., 2002; Garcia et al., 2005), in order to reduce the synchrony in STN population along with the minimal rise in the firing rate. In addition to exploring DBS parameters, a range of stimulus waveforms (such as rectangular monophasic (MP) and biphasic (BP) current pulses) and different types of stimulation configurations (such as single contact point (SCP), four contact points (FCP) and multiple contact points (MCP)) were also implemented (Figure 2) (Cogan, 2008; Lee et al., 2016).

In the present study, the current pulses which given to neuronal network are in the form of monophasic and biphasic waveforms. The monophasic current pulse (*P*_*MP*_) was generated as the following,

(26) $$P_{MP}(t)=\{\begin{array}{cc} \;A_{DBS}, & t_{k}\leq t<t_{k}+\delta_{DBS}\\ \;0, & \;else \end{array}$$

where, *t*_*k*_ are the onset times of the current pulses, *A*_*DBS*_ is the amplitude of the current pulse, δ_*DBS*_ is the current pulse width.

The biphasic current pulse (*P*_*BP*_) was generated as the following,

(27) $$P_{MP}(t)=\{\;\begin{array}{cc} A_{DBS}, & t_{k}\leq t<t_{k}+\frac{\delta_{DBS}}{2}\\ -A_{DBS}, & t_{k}+\frac{\delta_{DBS}}{2}\leq t<t_{k}+\delta_{DBS}\\ 0, & else \end{array}$$

where, *t*_*k*_ are the onset times of the current pulses, *A*_*DBS*_ is the amplitude of the current pulse, δ_*DBS*_ is the current pulse width.

The influence of stimulation on a particular neuron will depend on the position of the stimulation electrode in the neuronal network (Cogan, 2008). The effect of stimulation will decay as the distance between electrode position (*i*_*c*_, *j*_*c*_) and neuronal position (*i, j*) increased which was modeled as a Gaussian neighborhood (Mandali and Chakravarthy, 2016). We have assumed that the center of the electrode to be the mean of the Gaussian which coincides with the lattice position (*i*_*c*_, *j*_*c*_) and the spread of stimulus current was controlled by the width of the Gaussian (σ).

(28) $$I_{ij}^{DBS-STN}(t)=\sum_{\beta =1}^{N_{cp}^{x}}M_{\beta }(t)*P_{y}(t)*e^{\frac{-[{\left(i-i_{c}\right)}^{2}+{\left(j-j_{c}\right)}^{2}]}{\sigma_{DBS-STN}^{2}}}$$

where, $I_{ij}^{DBS-STN}\left(t\right)$ is the DBS current received by STN neuron at position (*i, j*) considering lattice position (*i*_*c*_, *j*_*c*_) as the electrode contact point at time *t*, $M_{\beta }(t)$ is the indicator function which controls the activation of stimulation site β, $N_{cp}^{x}$ is the number of activated stimulation contact points for different stimulation configurations *x* = [*SCP, FCP, MCP*] ($N_{cp}^{SCP}$= 1, $N_{cp}^{FCP}$= 4, $N_{cp}^{MCP}$= Number of neurons in simulated network - 1024), *P*_*y*_(*t*) is the current pulse at time *t* for *y* = [*MP, BP*], σ_*DBS*−*STN*_ is used to control the spread of stimulus current in STN network.

DBS was implemented in the proposed excitotoxicity model by the following criterion,

(29) $$I_{ij}^{DBS-STN}(N_{sc},t)=\{\begin{array}{cc} \;0, & N_{sc}(t)>N_{i}\\ I_{ij}^{DBS-STN}(t), & N_{sc}(t)\leq N_{i} \end{array}$$

where, $I_{ij}^{DBS-STN}\left(t\right)$ is the instantaneous change in the stimulation current to STN neuron at position (*i, j*) based on the number of surviving SNc neurons at time *t*, *N*_*sc*_(*t*) is the instantaneous number of surviving SNc neurons, *N*_*i*_ is the number representing SNc cell loss at which therapeutic intervention was employed.

#### 2.7.5. Antidromic Activation

The mechanism of how DBS alleviates advanced PD symptoms is not precise. One of the theories behind the therapeutic effect of DBS is activation of afferent connections of STN which results in antidromic activation of cortical, GPi or GPe neurons (Lee et al., 2004; McIntyre et al., 2004; Hammond et al., 2008; Montgomery and Gale, 2008; Kang and Lowery, 2014; Chiken and Nambu, 2015). In our study, we implemented the antidromic activation of GPe during DBS therapy. Antidromic activation was implemented similarly to Mandali and Chakravarthy (2016), where a percentage of DBS current given to STN neurons were given directly to GPe neurons. Similar to DBS applied to STN, external stimulation current was given to GPe neuron in the form of Gaussian distribution. The specifications of antidromic activation were described by the following equation,

(30) $$I_{ij}^{DBS-GPe}(t)=\sum_{\beta =1}^{N_{cp}^{x}}M_{\beta }(t)*P_{y}(t)*e^{\frac{-[{\left(i-i_{c}\right)}^{2}+{\left(j-j_{c}\right)}^{2}]}{\sigma_{DBS-GPe}^{2}}}$$

where, $I_{ij}^{DBS-GPe}\left(t\right)$ is the DBS current received by GPe neuron at position (*i, j*) considering lattice position (*i*_*c*_, *j*_*c*_) as the electrode contact point, $M_{\beta }(t)$ is the indicator function which controls the activation of stimulation site β, $N_{cp}^{x}$ is the number of activated stimulation contact points for different stimulation configurations *x* = [*SCP, FCP, MCP*] ($N_{cp}^{SCP}$= 1, $N_{cp}^{FCP}$= 4, $N_{cp}^{MCP}$= Number of neurons in simulated network - 1024), *P*_*y*_(*t*) is the current pulse at time *t* for *y* = [*MP, BP*], *A*_*DBS*−*GPe*_ is the portion of DBS current pulse amplitude given as antidromic activation to GPe neurons, σ_*DBS*−*GPe*_ is used to control the spread of stimulus current in GPe ensemble.

The DBS therapy with antidromic activation was implemented in the proposed excitotoxicity model by the following criterion,

(31) $$I_{ij}^{DBS-STN}(N_{sc},t)=\{\begin{array}{cc} 0, & N_{sc}(t)>N_{i}\\ I_{ij}^{DBS-STN-AA}(t), & N_{sc}(t)\leq N_{i} \end{array}$$

(32) $$I_{ij}^{DBS-GPe}(N_{sc},t)=\{\begin{array}{cc} \;0, & N_{sc}(t)>N_{i}\\ I_{ij}^{DBS-GPe}(t), & \;N_{sc}(t)\leq N_{i} \end{array}$$

where, $I_{ij}^{DBS-STN-AA}\left(t\right)$ is the DBS current received by STN neuron at position (*i, j*) considering lattice position (*i*_*c*_, *j*_*c*_) as the electrode contact point with antidromic activation $\left(A_{DBS-GPe}=Per_{AA}*A_{DBS-STN};A_{DBS-STN}^{\prime }=(1-Per_{AA})*A_{DBS-STN}\right),Per_{AA}$ , *Per*_*AA*_ is the proportion of *A*_*DBS*−*STN*_ applied as *A*_*DBS*−*GPe*_, $A_{DBS-STN}^{{}^{\prime }}$ is the portion of DBS current pulse amplitude given to STN neurons during antidromic activation, *N*_*sc*_(*t*) is the instantaneous number of surviving SNc neurons, *N*_*i*_ is the number representing SNc cell loss at which therapeutic intervention was employed.

#### 2.7.6. STN Axonal & Synaptic Failures

*In-vitro* recordings observed depression in the synapse of STN neurons with SNc and is believed to be due to the delivery of continuous high-frequency stimulation pulses (Ledonne et al., 2012). This synaptic depression caused by increased STN activity during DBS arises due to an amalgamation of axonal and synaptic failures in the STN (Shen and Johnson, 2008; Ammari et al., 2011; Moran et al., 2011, 2012; Zheng et al., 2011; Carron et al., 2013; Rosenbaum et al., 2014).

The effect of synaptic depression due to DBS of the STN was implemented by the following criterion,

(33) $$W_{STN\to SNc}\left(S_{DBS},t\right)=\{\begin{array}{cc} W_{STN\to SNc}, & S_{DBS}=OFF\\ W_{STN\to SNc}*W_{ASF}(Per_{ASF}), & S_{DBS}=ON \end{array}$$

where, $W_{STN\to SNc}(S_{DBS,\;t})$ is the instantaneous change in synaptic weight of STN to SNc based $S_{DBS}$ = {ON, OFF}, $S_{DBS}$ is DBS stimulation, *W*_*ASF*_ is the weight matrix based on the percentage of axonal and synaptic failures (*Per*_*ASF*_).

(34) $$W_{STN\to GPe}\left(S_{DBS},t\right)=\{\begin{array}{cc} W_{STN\to GPe}, & S_{DBS}=OFF\\ \text{​​}W_{STN\to GPe}*W_{ASF}(Per_{ASF}), & S_{DBS}=ON \end{array}$$

where, $W_{STN\to GPe}(S_{DBS,\;t})$ is the instantaneous change in synaptic weight of STN to GPe based $S_{DBS}$ = {ON, OFF}, $S_{DBS}$ is DBS stimulation, *W*_*ASF*_ is the weight matrix based on the percentage of axonal and synaptic failures (*Per*_*ASF*_).

### 2.8. Network Analysis

We analyzed the dynamics of the network (STN-GPe-SNc) by firing frequency (Dayan and Abbott, 2005), network synchrony (Pinsky and Rinzel, 1995) and bursting measures (van Elburg and van Ooyen, 2004). The equations used to compute these measures are described below.

#### 2.8.1. Frequency of Firing

The spike-count firing rate is the measure of the number of action potentials for a given duration of time (Dayan and Abbott, 2005). The instantaneous mean firing rate $\left(r_{ij}^{x}\left(t\right)\right)$ of a neuron at lattice position (*i, j*) at time *t* was calculated with a fixed sliding window Δ*t* (0.1 s) which is similarly to Equations (17), (18). The mean firing rate of the population of neurons is simply the average of instantaneous mean firing rate across the number of neurons and the simulation time.

#### 2.8.2. Synchronization

Neuronal synchronization is the measure of synchronicity (high synchrony - almost all neurons firing at once, low synchrony - least number of neurons firing at once) in the population of neurons within a network (Golomb, 2007). We had quantified the synchrony in the population of neurons at time *t* by following equation (Pinsky and Rinzel, 1995),

(35) $$\begin{array}{r} R_{x}\left(t\right)=\frac{1}{N\;*\;e^{i*\theta \left(t\right)}}\overset{N}{\underset{j=1}{\sum }}e^{i*\phi_{j}\left(t\right)} \end{array}$$

(36) $$\begin{array}{r} \phi_{j}\left(t\right)=2*\pi *\frac{\left(T_{j,k}\;-\;t_{j,k}\right)}{t_{j,k+1}\;-\;t_{j,k}} \end{array}$$

where, *R*_*x*_(*t*) is the instantaenous synchronization measure (0 ≤ *R*_*x*_(*t*) ≤ 1), *x* being GPe or SNc or STN neuron, *N* is the number of neurons in the network, θ(*t*) is the instantaneous average phase of neurons, ϕ_*j*_(*t*) is the instantaneous phase of jth neuron, *t*_*j, k*_ and *t*_*j, k*+1_ are the spike times of *k*th and (*k*+1)th spike of *j*th neuron, respectively, *T*_*j, k*_∈[*t*_*j, k*_, *t*_*j, k*+1_].

#### 2.8.3. Bursting

If a neuron fires repeatedly with discrete groups of spikes, this dynamic state is termed as burst. Between two bursts, there is a period of quiescence where there will be no spikes. Burst can have two (doublet), three (triplet), four (quadruplet) or many spikes in it (Izhikevich, 2006). We had quantified the bursting of a neuron at lattice position (*i, j*) across time by following equation (van Elburg and van Ooyen, 2004),

(37) $$\begin{array}{r} B_{i,j}=\frac{2\;*\;Var\left(t_{i,j,k+1}\;-\;t_{i,j,k}\right)\;-\;Var\left(t_{i,j,k+2}\;-\;t_{i,j,k}\right)}{2*E^{2}\left(t_{i,j,k+1}\;-\;t_{i,j,k}\right)} \end{array}$$

where, *B*_*i, j*_ is the measure of bursting of a neuron at lattice position (*i, j*), *Var* is the variance of the spike times, *E* is the expected value (mean) of the spike times, *t*_*i, j, k*_, *t*_*i, j, k*+1_ and *t*_*i, j, k*+2_ are the spike times of *k*th, (*k*+1)th and (*k*+2)th spike of a neuron at lattice position (*i, j*), respectively.

## 3. Results

**Figure 3:**
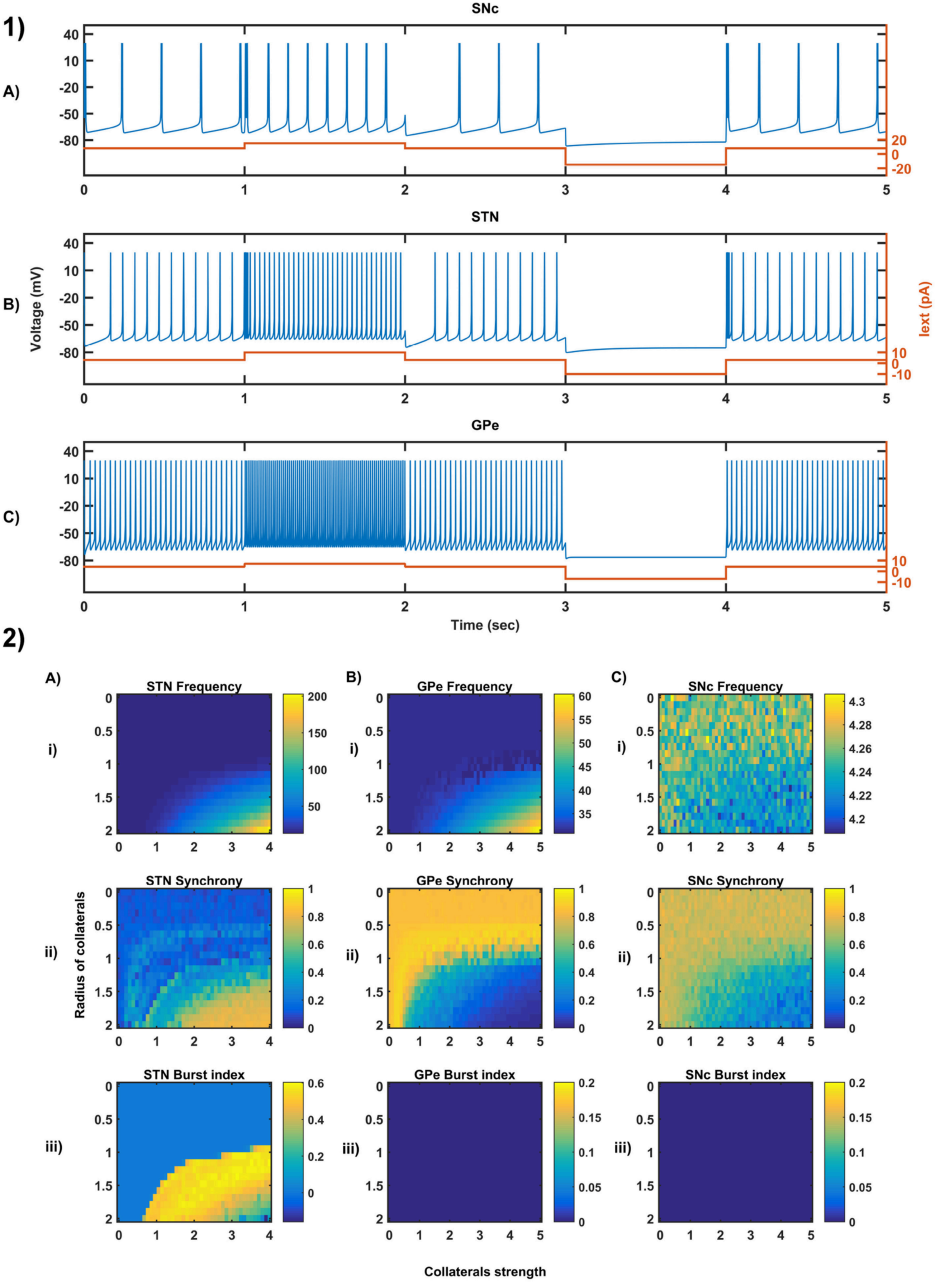
Characteristic behavior in the single-neuron and the population of neurons of different neuronal types. **(1)** Characteristics firing patterns of SNc **(A)**, STN **(B)**, and GPe **(C)** for varying external currents (orange line - current in picoAmpere (pA)). **(2)** The response of STN **(A)**, GPe **(B)**, and SNc **(C)** populations for varying lateral connection strength (*A*_*x*_) and radius (*R*_*x*_) at the level of network properties [Frequency **(i)**, Synchrony **(ii)**, Burst Index **(iii)**]. *I*_*ext*_, External current applied; STN, SubThalamic Nucleus; SNc, Substantia Nigra pars compacta; GPe, Globus Pallidus externa.

**Figure 4:**
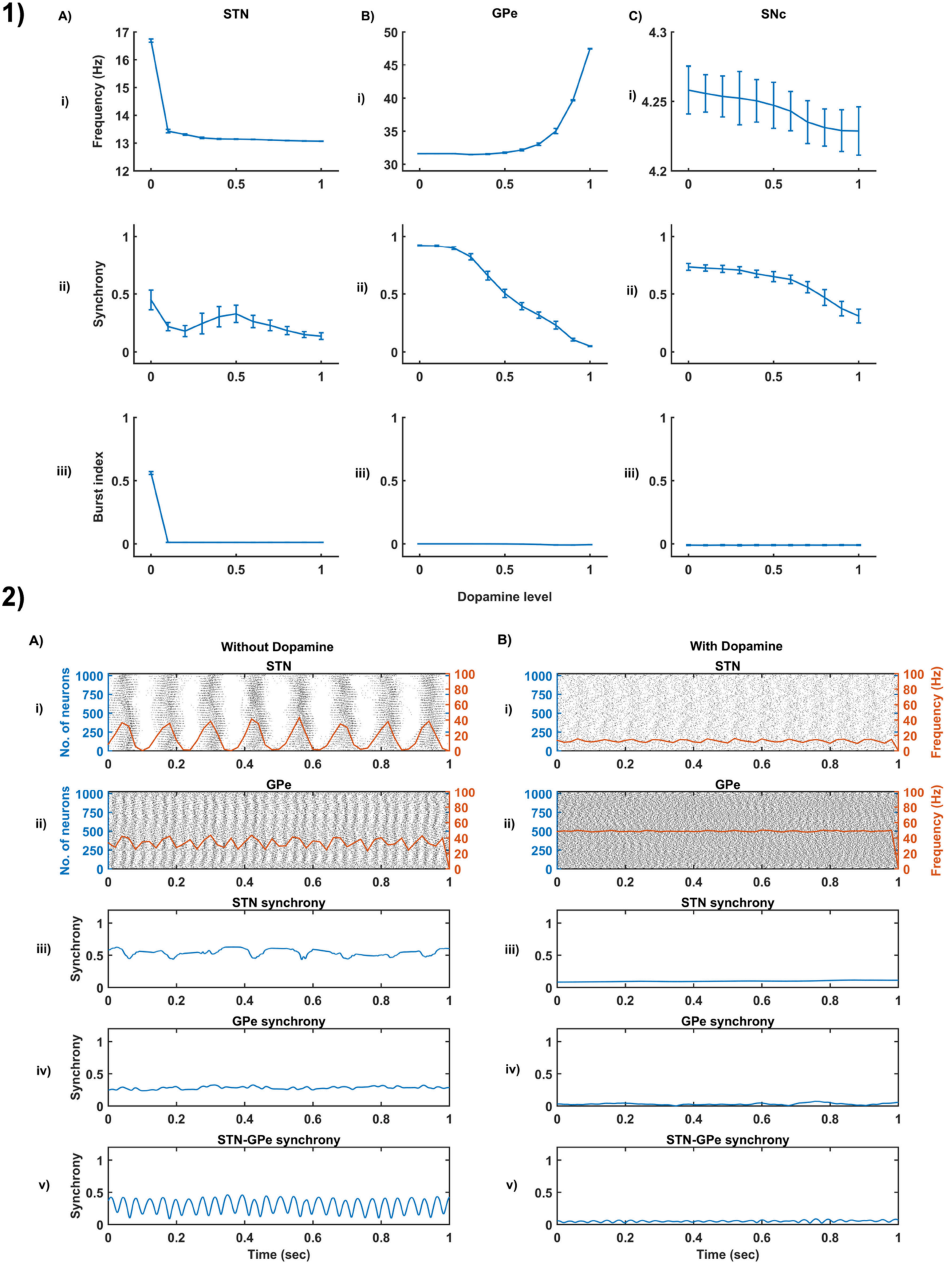
Dopamine effect on the basal activity of different neuronal types. **(1)** The response of STN **(A)**, GPe **(B)**, and SNc **(C)** populations for varying dopamine levels at the level of network properties [Frequency **(i)**, Synchrony **(ii)**, and Burst Index **(iii)**]. **(2)** The response of STN-GPe network without **(A)** & with **(B)** dopamine - Raster plots of STN **(i)** & GPe **(ii)** populations overlaid with spike-count firing rate (orange line), Synchrony plots of STN **(iii)**, GPe **(iv)**, and combined STN-GPe **(v)**. STN, SubThalamic Nucleus; SNc, Substantia Nigra pars compacta; GPe, Globus Pallidus externa.

We have investigated the Izhikevich parameters of STN, SNc and GPe which were chosen from the literature (Michmizos and Nikita, 2011; Cullen and Wong-Lin, 2015; Mandali et al., 2015) for their characteristic firing pattern and other biological properties (Figure 3-1). We then extensively studied the effect of lateral connections in the network of neurons (Figure 3-2). Next, we have explored the effect of dopamine on the network of GPe, SNc, and STN neurons and compared with published data (Figure 4).

**Figure 5:**
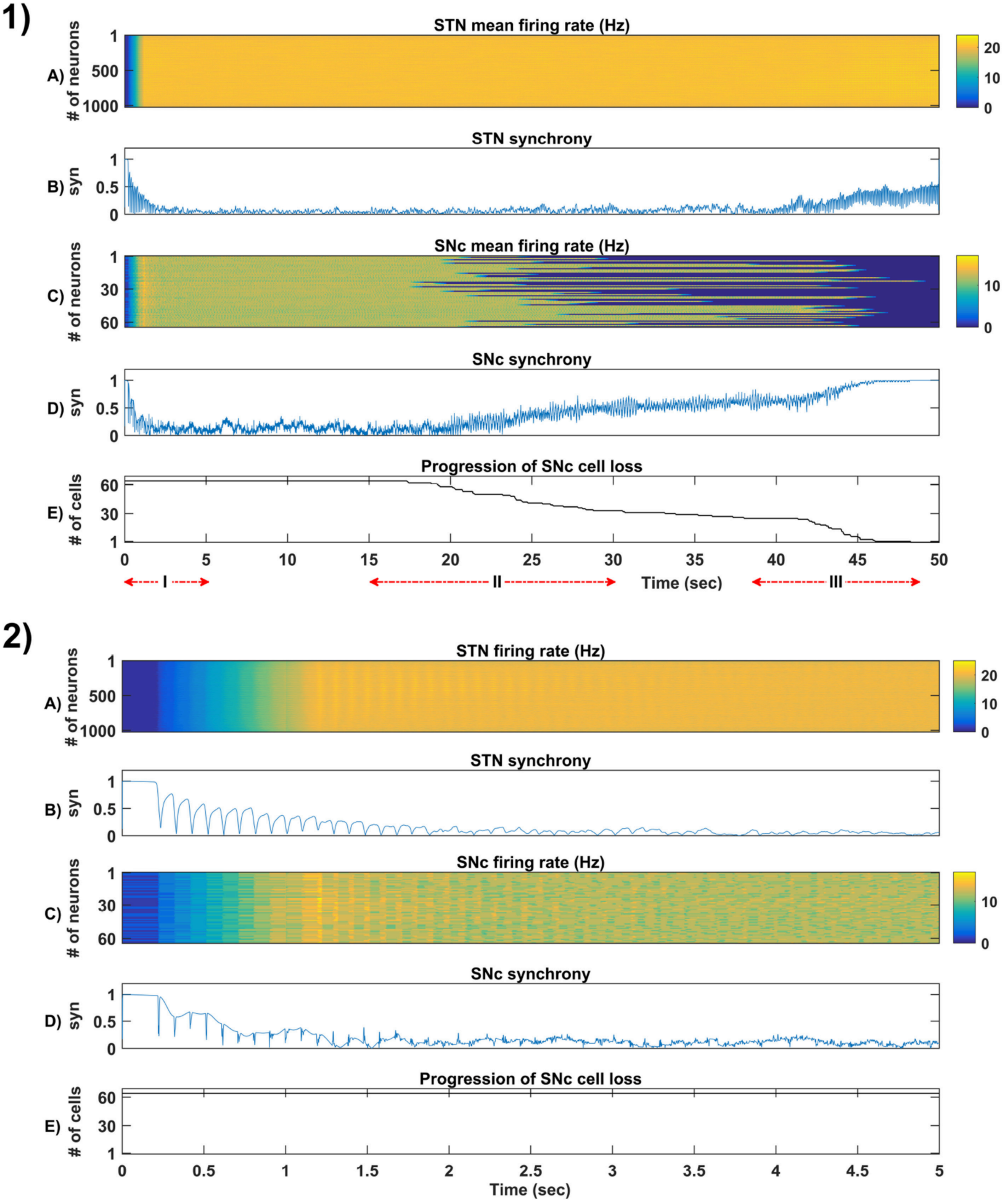
Simulation plots of whole and step-wise mechanism (I) of the proposed excitotoxicity model. **(1)** Whole 50 sec simulation plots of the proposed excitotoxicity model. **(2)** Part-I of **(1)** Simulation plots of STN-SNc loop dynamics - Mean firing rate (1 s) of STN **(A)** & SNc **(C)**, Synchrony (syn) of STN **(B)** & SNc **(D)**, Progression of SNc cell loss **(E)**. STN, SubThalamic Nucleus; SNc, Substantia Nigra pars ompacta; GPe, Globus Pallidus externa.

**Figure 6:**
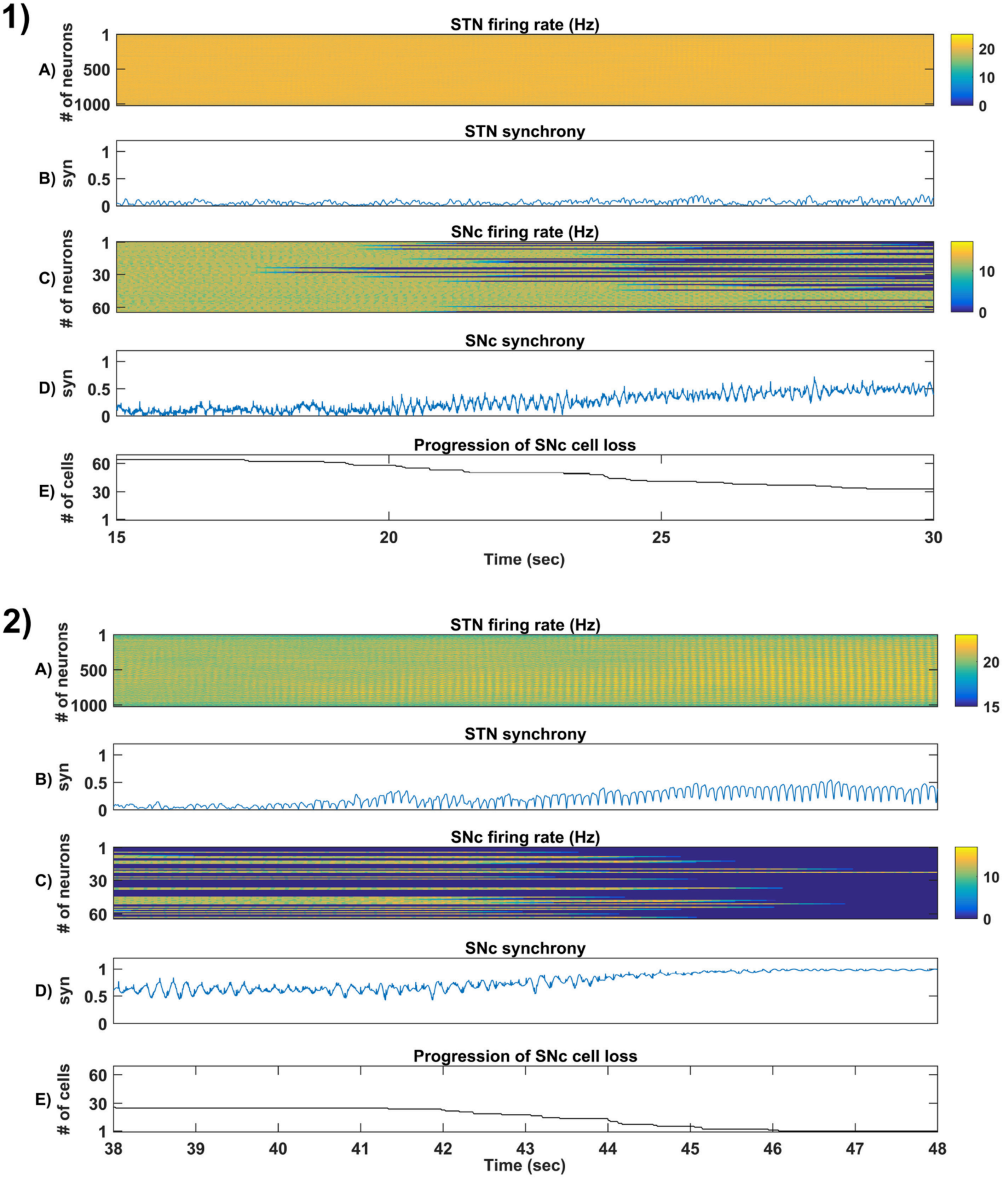
Simulation plots of step-wise mechanisms (II, III) of the proposed excitotoxicity model. **(1)** Part-II of (Figure 5-1) Stress-induced neurodegeneration in SNc. **(2)** Part-III of (Figure 5-1) STN-mediated runaway effect of neurodegeneration in SNc - Mean firing rate (1 s) of STN **(A)** & SNc **(C)**, Synchrony (syn) of STN **(B)** & SNc **(D)**, Progression of SNc cell loss **(E)**. STN, SubThalamic Nucleus; SNc, Substantia Nigra pars compacta; GPe, Globus Pallidus externa.

**Figure 7:**
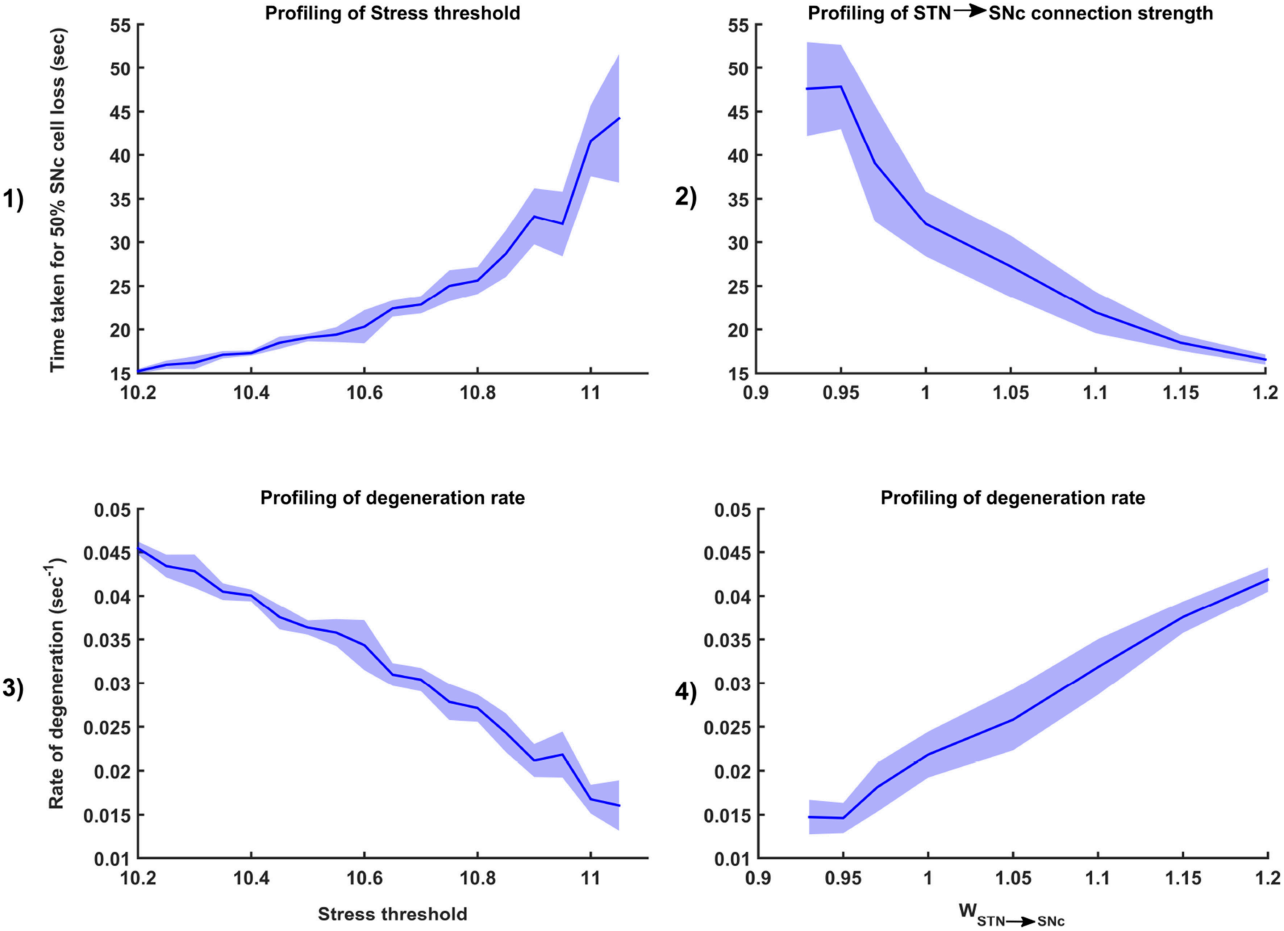
Sensitivity of the proposed model toward parameter uncertainty. Time taken for 50% SNc cell loss for varying stress threshold (*Q*_*thres*_) **(1)** and connection strength from STN → SNc (*W*_*STN* → *SNc*_) **(2)**. Rate of degeneration (λ) for varying stress threshold (*Q*_*thres*_) **(3)** and connection strength from STN → SNc (*W*_*STN* → *SNc*_) **(4)**. STN, SubThalamic Nucleus; SNc, Substantia Nigra pars compacta.

**Figure 8:**
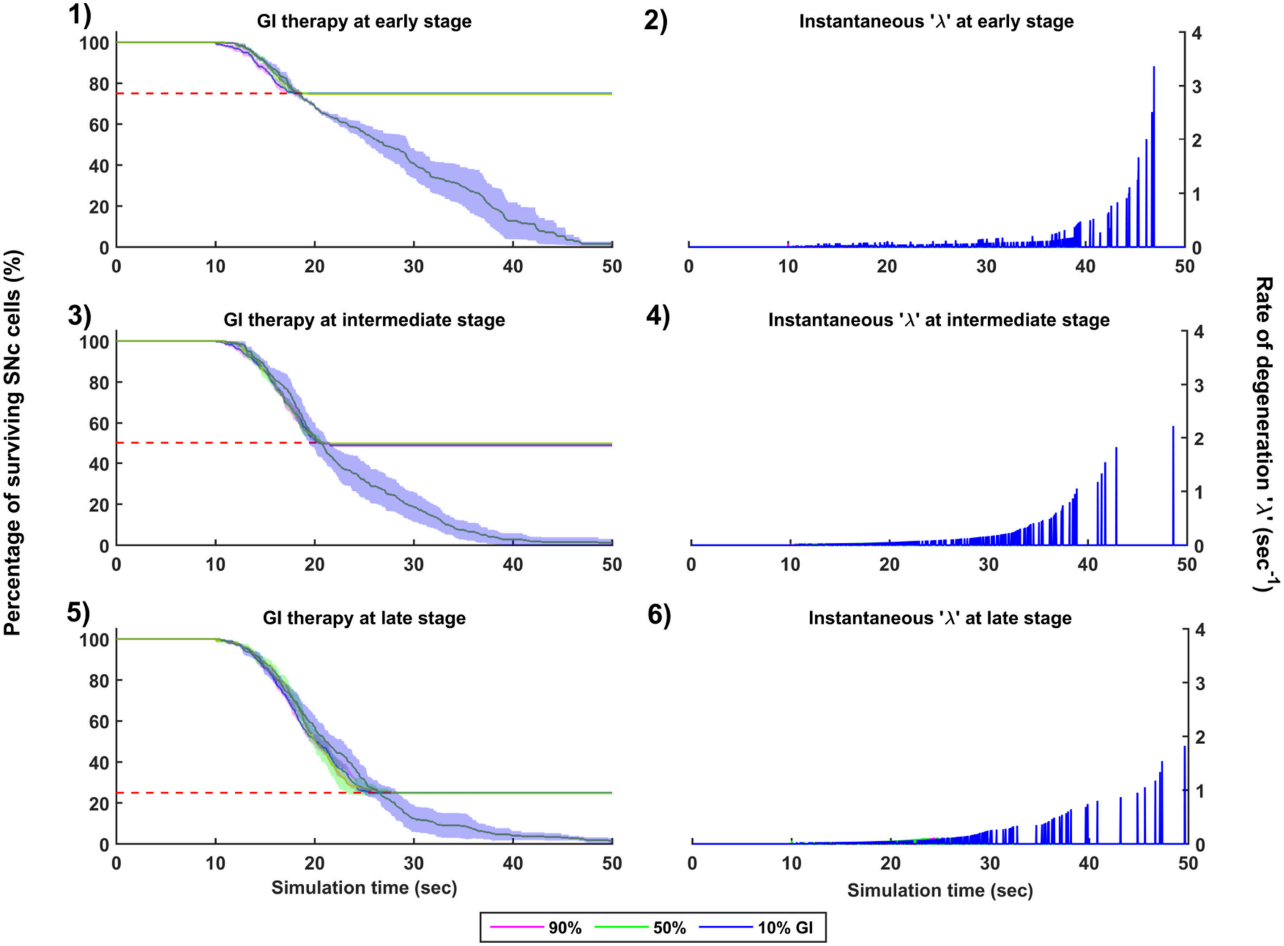
Simulation plots for Glutamate Inhibition (GI) therapy. Progression of SNc cell loss for 90, 50, and 10% GI at early (25%) **(1)**, intermediate (50%) **(3)** and late (75%) **(5)** stages of SNc cell loss. Instantaneous rate of degeneration (λ) for 90, 50, and 10% GI at early (25%) **(2)**, intermediate (50%) **(4)** and late (75%) **(6)** stages of SNc cell loss. SNc, Substantia Nigra pars compacta.

**Figure 9:**
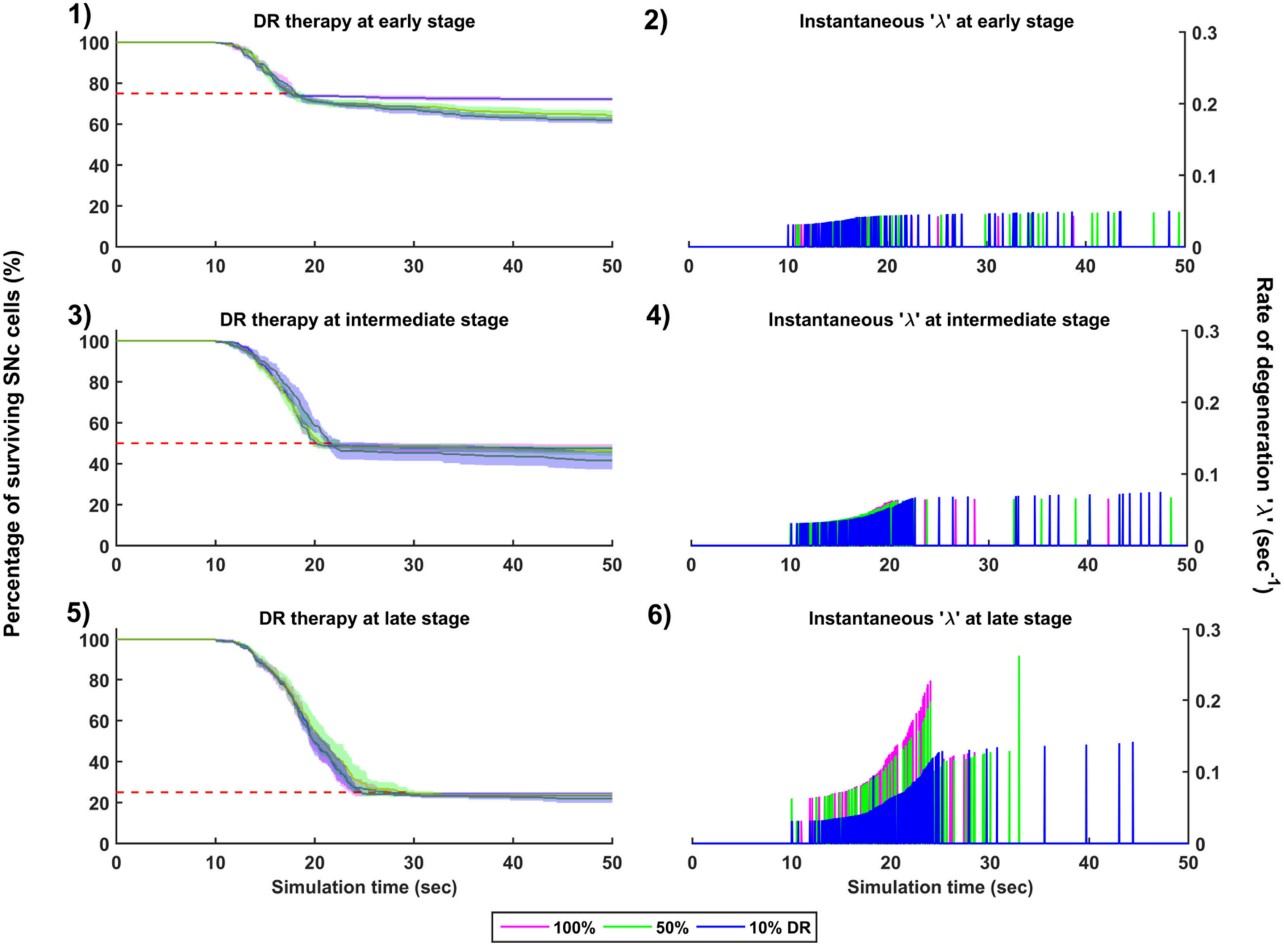
Simulation plots for Dopamine Restoration (DR) therapy. Progression of SNc cell loss for 100, 50, and 10% DR at early (25%) **(1)**, intermediate (50%) **(3)** and late (75%) **(5)** stages of SNc cell loss. Instantaneous rate of degeneration (λ) for 100, 50, and 10% DR at early (25%) **(2)**, intermediate (50%) **(4)** and late (75%) **(6)** stages of SNc cell loss. SNc, Substantia Nigra pars compacta.

**Figure 10:**
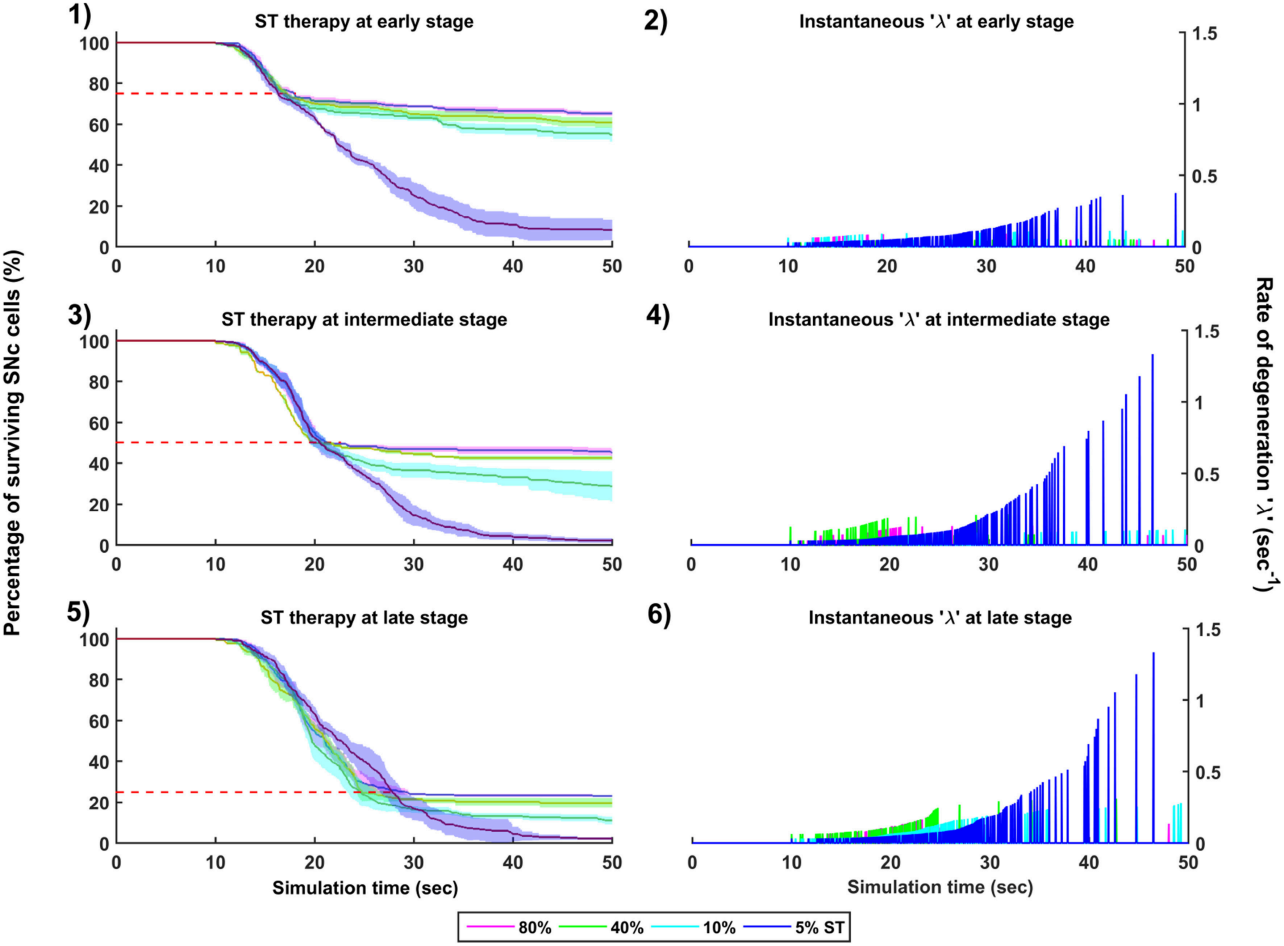
Simulation plots for SuthalamoTomy (ST) therapy. Progression of SNc cell loss for 80, 40, 10, and 5% ST at early (25%) **(1)**, intermediate (50%) **(3)** and late (75%) **(5)** stages of SNc cell loss. Instantaneous rate of degeneration (λ) for 80, 40, 10, and 5% GI at early (25%) **(2)**, intermediate (50%) **(4)** and late (75%) **(6)** stages of SNc cell loss. SNc, Substantia Nigra pars compacta.

**Figure 11:**
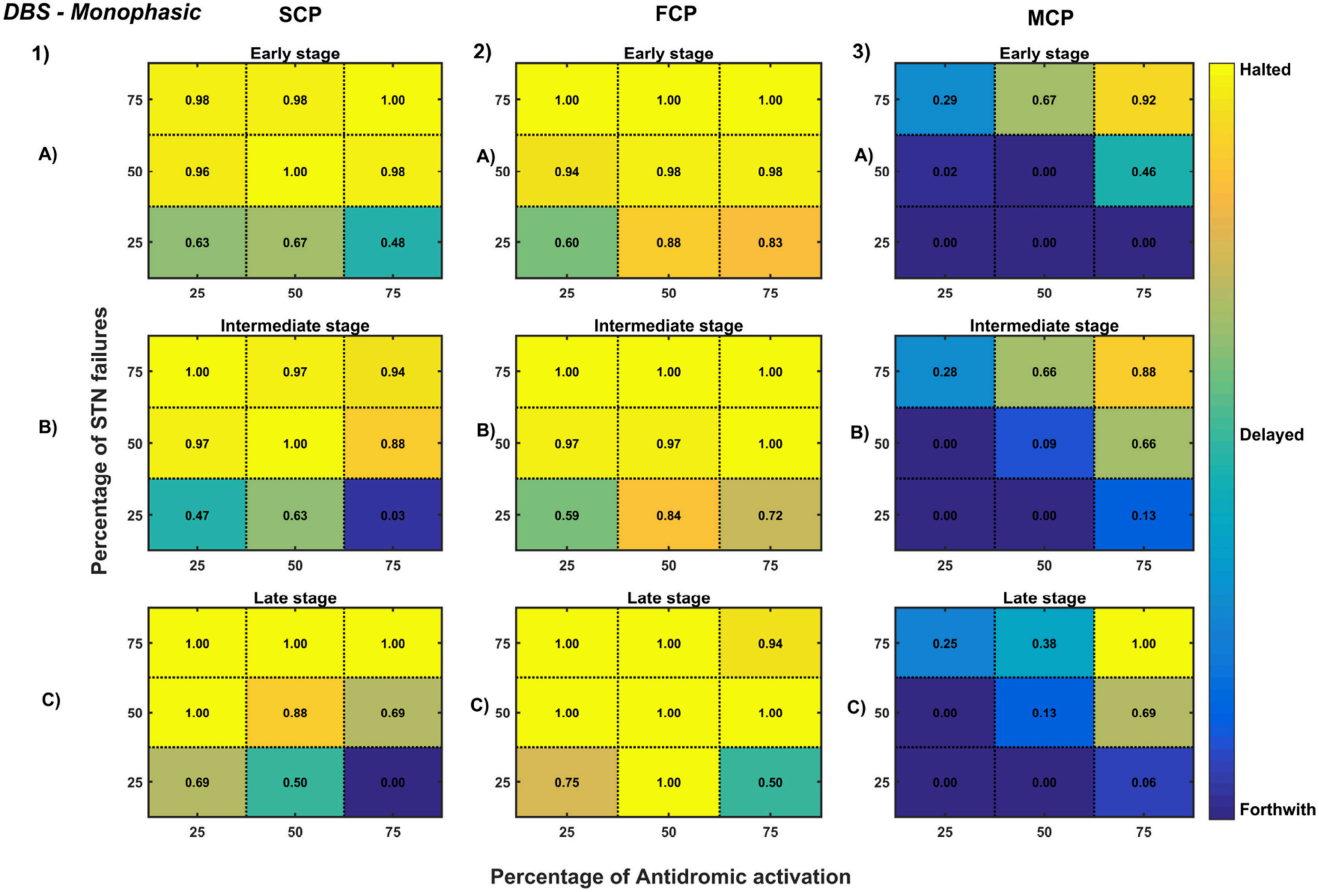
Simulation plots for Monophasic DBS therapy. Profiling of monophasic stimulus waveform for different stimulation configuration in order to achieve the maximal neuroprotective effect of DBS. Confusion matrices for SCP **(1)**, FCP **(2)**, and MCP **(3)** configurations showing survival ratios of SNc cells for different percentage activation of antidromic activation and STN axonal & synaptic failures at early (25%) **(A)**, intermediate (50%) **(B)** and late (75%) **(C)** stages of SNc cell loss. Ratios around 0 is indicated as forthwith (indigo), ratios around 0.5 is indicated as delayed (light green), and ratios around 1 is indicated as halted (yellow). DBS, Deep Brain Stimulation; SNc, Substantia Nigra pars compacta; STN, SubThalamic Nucleus; SCP, Single Contact Point; FCP, Four Contact Point; MCP, Multiple Contact Point.

**Figure 12:**
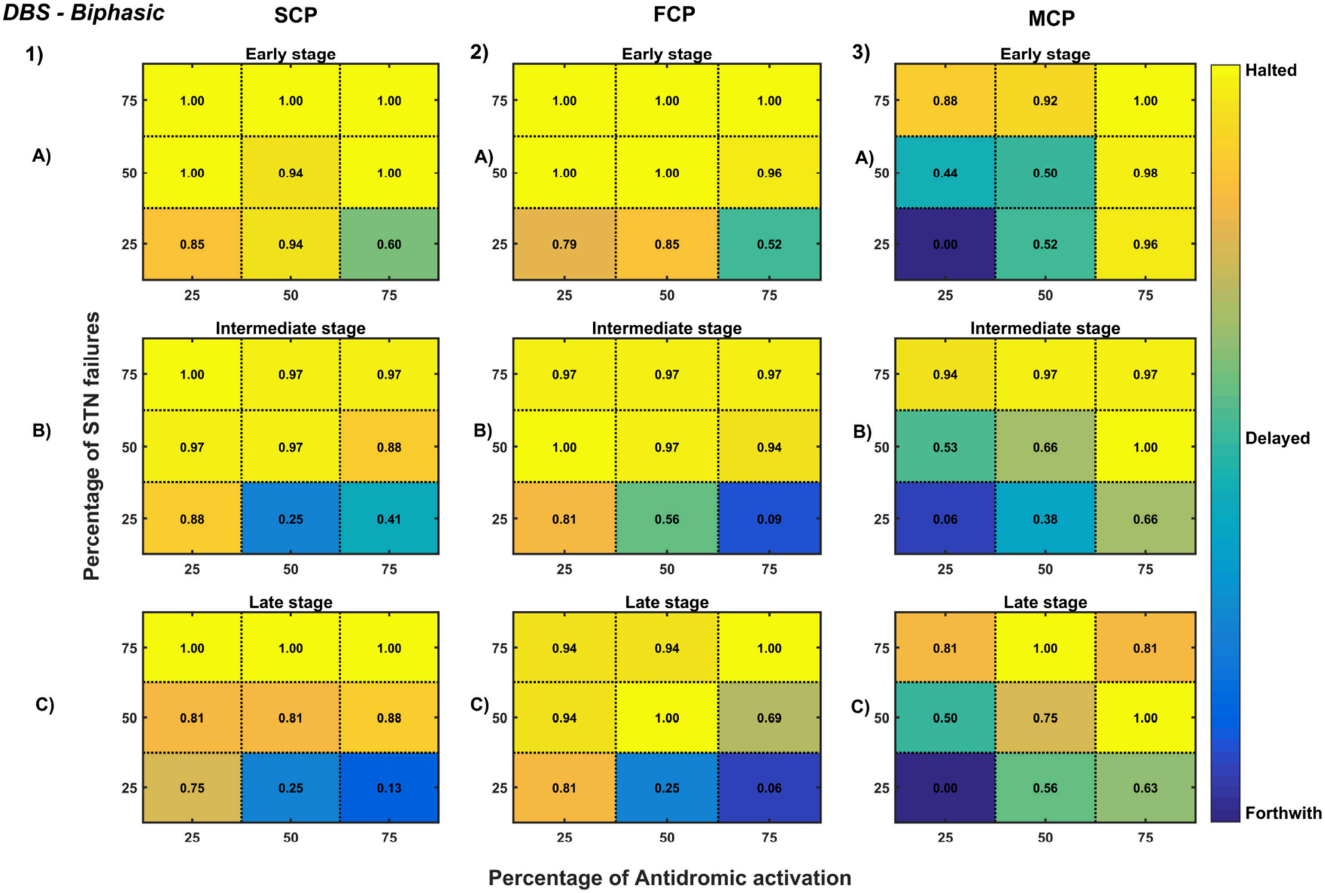
Simulation plots for Biphasic DBS therapy. Profiling of biphasic stimulus waveform for different stimulation configuration in order to achieve the maximal neuroprotective effect of DBS. Confusion matrices for SCP **(1)**, FCP **(2)**, and MCP **(3)** configurations showing survival ratios of SNc cells for different percentage activation of antidromic activation and STN axonal & synaptic failures at early (25%) **(A)**, intermediate (50%) **(B)** and late (75%) **(C)** stages of SNc cell loss. Ratios around 0 is indicated as forthwith (indigo), ratios around 0.5 is indicated as delayed (light green), and ratios around 1 is indicated as halted (yellow). DBS, Deep Brain Stimulation; SNc, Substantia Nigra pars compacta; STN, SubThalamic Nucleus; SCP, Single Contact Point; FCP, Four Contact Point; MCP, Multiple Contact Point.

Then, we showed the results of the proposed excitotoxicity model which exhibits STN-mediated excitotoxicity in SNc (Figures 5, 6) and studied their sensitivity to parameter uncertainty (Figure 7). Finally, we have explored current therapeutics such as glutamate inhibition (Figure 8), dopamine restoration (Figure 9), subthalamotomy (Figure 10) and deep brain stimulation (Figures 11, 12) which might have a neuroprotective effect on the progression of SNc cell loss.

### 3.1. Characteristic Firing of Different Neuronal Types

The firing response of a single neuron to different external current input was characterized for the three different neuronal types involved in the excitotoxicity model (Figure 3-1). In the proposed model, we adjusted $I_{ij}^{x}$ and other parameters of the Izhikevich model such that the basal firing frequencies of the different neuronal types match with experimental data (Modolo et al., 2007; Tripathy et al., 2014). The adjusted values can be seen in the Table 1.

The SNc neurons experimentally exhibit two distinct firing patterns: low-frequency irregular tonic or background firing (3–8 Hz) and high-frequency regular phasic or burst firing (~20 Hz) (Grace and Bunney, 1984a,b). The Izhikevich parameters which were chosen for SNc neurons configured the model to exhibit both types of firing patterns. Other properties such as doublet-spikes which were occasionally observed experimentally (Grace and Bunney, 1983) were also exhibited (Figure 3-1A). In the present model, SNc neuron basal firing rate were required to be ~4 Hz which is in the range of 3–8 Hz observed experimentally (Grace and Bunney, 1984a). Similar to SNc, STN neurons also exhibit tonic pacemaking firing and phasic high-frequency bursting (Beurrier et al., 1999; Allers et al., 2003). The basal firing rate of STN neurons was required to be ~13 Hz which is in the range of 6–30 Hz observed experimentally (Allers et al., 2003; Lindahl et al., 2013). The STN neurons also exhibit characteristic inhibitory rebound which was observed experimentally (Figure 3-1B) (Hamani et al., 2004; Johnson, 2008). Unlike SNc and STN, GPe neurons exhibit high-frequency tonic firing which was interpreted by bursts and pauses (Kita and Kita, 2011; Hegeman et al., 2016). The Izhikevich parameters which were chosen for GPe neurons were able to exhibit high-frequency firing without any bursts (Figure 3-1C). The basal firing rate of GPe neurons was required to be ~30 Hz which is in the range of 17–52 Hz observed experimentally (Lindahl et al., 2013).

### 3.2. Behavior Regimes With Varying Collateral Strength and Radius

We now study the network dynamics of each of the three neuronal types in a 2D array with lateral connections. The effect of network structural properties such as the strength and neighborhood size of the lateral connections on the network functional properties such as average firing rate, network synchrony, and burst index was studied (Figure 3-2). The suitable values of lateral connection strength and radius for each neuronal type were chosen in correlation with experimental data (Humphries et al., 2006; Tepper and Lee, 2007). The selected values can be seen in the Table 1. As specified above, $I_{ij}^{x}$, *A*_*x*_ and *R*_*x*_ was adjusted such that the basal population activity correlated well with the experimental data (Humphries et al., 2006; Tepper and Lee, 2007).

The network dynamics of STN plays a vital role in the proposed model of excitotoxicity, in this scenario we have studied the role of lateral connections in regulating STN network properties. The basal STN population activity without lateral connections showed regular spiking without any bursting type of behavior. Contrarily, the basal STN population activity with lateral connections showed the bursting type of activity (not shown here).

### 3.3. Dopamine Effect on the Basal Activity of Different Neuronal Populations

From the simulated results, it is clear that as DA level increases the mean firing rate decreases in STN, increases in GPe and decreases in SNc (Figure 4-1). The network synchrony decreases in all neuronal populations as DA levels increases. However, in the case of STN, the decrease is not monotonic (Figure 4-1Aii) where high synchrony was observed at moderate levels of DA, with synchrony falling on either side. This high synchronicity at moderate levels of DA is a result of the change in firing pattern from asynchronous bursting to synchronous spiking which can be correlated with burst index (Figure 4-1Aiii) in STN population. In the dopamine-depleted condition, STN shows the bursting type of firing pattern which was exhibited by our model consistent with published studies (Vila et al., 2000; Ammari et al., 2011; Park et al., 2015). The following trend of STN activity was observed when DA level increases from 0 to 1: synchronous bursting, asynchronous bursting, synchronous spiking and asynchronous spiking. At very low DA levels (0–0.1), the STN exhibits regular bursting (Figure 4-1Aiii) with high synchrony (Figure 4-1Aii). At low DA levels (0.1–0.3), the STN exhibits an irregular mixed mode of bursting and singlet-spiking with low synchrony (Figure 4-1Aii). At moderate DA levels (0.3–0.7), the STN exhibits regular singlet-spiking (Figure 4-1Aiii) with high synchrony (Figure 4-1Aii). Moreover, at high DA levels (0.3–1), the STN exhibits irregular singlet-spiking with low synchrony (Figure 4-1Aii).

STN-GPe dynamics is known to play an important role in PD pathological oscillations that are thought to be strongly related to the cardinal symptoms of PD (Bergman et al., 1994; Brown, 2003; Litvak et al., 2011; Park et al., 2011). Numerous computational models were developed to explain the pathological oscillations in STN-GPe (Terman et al., 2002; Pavlides et al., 2015; Shouno et al., 2017). The connectivity pattern between STN and GPe was explored by using a conductance-based model (Terman et al., 2002) which exhibited different rhythmic behaviors. In our model, the connectivity pattern between STN and GPe was considered to be dopamine-dependent (Cragg et al., 2004; Mandali et al., 2015) and spontaneous activity of the STN-GPe network was studied with no external input current. Under normal DA conditions, low synchrony and minimal oscillations were exhibited by the STN-GPe network (Figure 4-2B) (Kang and Lowery, 2013). It was reported that dopamine-depleted condition results in pathological oscillations in STN characterized by high synchrony and beta range oscillations (Brown et al., 2001; Weinberger et al., 2006; Park et al., 2010, 2011; Lintas et al., 2012; Kang and Lowery, 2013; Pavlides et al., 2015). In our model during dopamine-depleted conditions, high synchrony and the higher rate of oscillations were exhibited in the STN-GPe network, and beta range oscillations were also observed in STN population (Figure 4-2A).

### 3.4. STN-Induced Excitotoxicity in SNc

The proposed excitotoxicity model was able to exhibit STN-mediated excitotoxicity in SNc which was precipitated by energy deficiency (Albin and Greenamyre, 1992; Beal et al., 1993; Greene and Greenamyre, 1996; Rodriguez et al., 1998; Blandini, 2001, 2010; Ambrosi et al., 2014) (Figures 5, 6). For a more detailed explanation of the excitotoxicity results obtained, we have sub-divided 50 s simulation into three parts - (I) STN-SNc loop dynamics (normal condition), (II) Stress-induced neurodegeneration in SNc (pre-symptomatic PD condition), and (III) STN-mediated runaway effect of neurodegeneration in SNc (symptomatic PD condition).

#### 3.4.1. (I) STN-SNc Loop Dynamics

In the first part of the simulation, connectivity between STN and SNc were introduced at *t* = 0, and the model exhibited decreased synchrony in STN and SNc over time (Figure 5-2B). The results showed the pivotal role of dopamine in modulating STN activity (Cragg et al., 2004; Lintas et al., 2012; Yang et al., 2016). The excitatory drive from STN to SNc results in decreased synchrony in SNc due to increased inhibitory drive from lateral connections (Figure 5-2D). During this whole process, the stress threshold (*Q*_*thres*_ = 11.3) was fixed, and there was no SNc cell loss due to stress (Figure 5-2E).

#### 3.4.2. (II) Stress-Induced Neurodegeneration in SNc

In the second part of the simulation, stress threshold was slightly reduced from *Q*_*thres*_ = 11.3 to *Q*_*thres*_ = 10.8 at *t* = 10*s* to replicate PD-like condition in the model where stress-induced neurodegeneration gets initiated. The model exhibited stress-induced neurodegeneration in SNc where SNc cells start dying when stress variable $\left(Q_{ij}^{x}\right)$ exceeds the stress threshold (*Q*_*thres*_) which acts like an apoptotic threshold (Figure 6-1E). It was observed that there was no increased synchrony in the STN population as a result of SNc cell loss (Figure 6-1B). However, there was increased synchrony in the SNc population (Figure 6-1D) which might be due to reduced inhibitory drive from lateral connections as a result of SNc cell loss.

#### 3.4.3. (III) STN-Mediated Runaway Effect of Neurodegeneration in SNc

In the third part of the simulation, no parameters were changed, but after *t* = 40*s*, there was a rise in STN synchrony as a result of stress-induced SNc cell loss (Figure 6-2). A substantial amount of SNc cell loss (more than 50%) resulted in increased synchrony (Figure 6-2B) and firing rates (Figure 6-2A) of the STN population. As the STN synchrony increased, runaway effect kicks in where increased STN excitatory drive to SNc cells result in hastening the stress-induced neurodegeneration of remaining SNc cells (Figure 6-2E).

### 3.5. Sensitivity of Excitotoxicity Model Toward Parameter Uncertainty

To check the sensitivity of excitotoxicity model for different parametric values, we have considered two factors which can maximally influence the output results. Firstly, stress threshold (*Q*_*thres*_) which is analogous to the apoptotic threshold and is assumed to be dependent on the amount of available energy to the cell (Albin and Greenamyre, 1992; Greene and Greenamyre, 1996). Secondly, the synaptic weight between STN and SNc (*W*_*STN* → *SNc*_) which is analogous to synaptic modification and is assumed to be modulated by the excitatory drive from STN to SNc (Hasselmo, 1994, 1997).

#### 3.5.1. Stress Threshold (*Q*_*thres*_)

Simulation results showed that the time taken for 50% SNc cell loss (*t*_1/2_) increases as the stress threshold increases (Figure 7-1). The rate of degeneration or degeneration constant (λ) is the ratio of the number of SNc cells that degenerate in a given period of time compared with the total number of SNc cells present at the beginning of that period. The rate of degeneration (λ) decreases as the stress threshold increases (Figure 7-3). These results show the importance of stress threshold in regulating excitotoxic damage to SNc and also support the idea of “weak excitotoxicity hypothesis” where SNc cells showed increased susceptibility to glutamate due to impaired cellular energy metabolism (Albin and Greenamyre, 1992; Greene and Greenamyre, 1996).

#### 3.5.2. STN-SNc Synaptic Weight (*W*_*STN* → *SNc*_)

Simulation results showed that time taken for 50% SNc cell loss (*t*_1/2_) decreases as the STN-SNc synaptic weight increases (Figure 7-2). The rate of degeneration (λ) increases as the STN-SNc synaptic weight increases (Figure 7-4). These results show the extent of STN influence in the causation of excitotoxicity in SNc. They also support the notion that STN-mediated excitotoxicity might play a major role in SNc cell loss in PD condition (Rodriguez et al., 1998; Blandini, 2001, 2010; Ambrosi et al., 2014).

### 3.6. Strategies for Neuroprotection of SNc

We now extend the proposed excitotoxic model to study the effect of various therapeutic interventions on the progression of SNc cell loss. The following three types of interventions which were simulated: (1) drugs, (2) surgical interventions, and (3) Deep Brain Stimulation (DBS).

#### 3.6.1. Glutamate Inhibition Therapy

The effect of glutamate agonists and antagonists on the progression of SNc cell loss was implemented in the manner specified in the methods section. The onset of glutamate therapy at different stages of SNc cell loss showed that cell loss was delayed or halted (Figure 8). For the glutamate therapy which is initiated at 25, 50, and 75% SNc cell loss, the progression of SNc cell loss was halted when the percentage of glutamate inhibition administrated was above 50%. As the glutamate dosage increases the progression of SNc cell loss delays and after a particular dosage of glutamate inhibitors the SNc cell loss halts. There was no change in the course of SNc cell loss for low levels of glutamate inhibition (Figures 8-1, 8-3, 8-5). The peak of the instantaneous rate of degeneration decreases as the therapeutic intervention is delayed in the case of 10% glutamate inhibition (Figures 8-2, 8-4, 8-6).

#### 3.6.2. Dopamine Restoration Therapy

The effect of dopamine agonists on the progression of SNc cell loss was also implemented in the manner specified in the methods section. The onset of dopamine agonist therapy at different stages of SNc cell loss showed that the progression of cell loss was only delayed (Figure 9). For the dopamine agonists therapy which is initiated at 25, 50, and 75% SNc cell loss, the progression of SNc cell loss was delayed when the percentage of dopamine restoration was a mere 10%. The neuroprotective effect of dopamine agonist therapy is dependent on the level of restoration of dopamine tone on the STN. In other words, as the dopamine content in STN increases, the progression of SNc cell loss delays. Unlike glutamate inhibition, the progression of SNc cell loss was not halted even at 100% dopamine restored in all the case of intervention (Figures 9-1, 9-3, 9-5). The dopamine restoration therapy did not have much effect on the instantaneous rate of degeneration (Figures 9-2, 9-4, 9-6).

#### 3.6.3. Subthalamotomy

The effect of subthalamotomy on the progression of SNc cell loss was implemented in a way described in the methods section. The onset of STN ablation therapy at different stages of SNc cell loss showed that progression of cell loss was delayed or halted (Figure 10). The neuroprotective effect of subthalamotomy is dependent on the proportion of lesioning of STN population. In other words, as the proportion of STN lesioning increases the progression of SNc cell loss delays and halts only when almost all of the STN population is lesioned (Figures 10-1, 10-3, 10-5). The progression of SNc cell loss is halted only at 100% STN lesioning in all cases of intervention (not shown here). However, as the proportion of STN lesioning decreases, the rate of degeneration increases. Similarly to dopamine restoration therapy, subthalamotomy also did not have much effect on the instantaneous rate of degeneration (Figures 10-2, 10-4, 10-6).

#### 3.6.4. Deep Brain Stimulation of STN

The effect of deep brain stimulation on the progression of SNc cell loss was implemented in the way described in the methods section. Along with the stimulation of STN, the inhibitory drive to STN through the afferent connections as result of antidromic activation of the GPe population and the synaptic depression in STN as result of increased axonal and synaptic failures in STN were incorporated in the model.

As specified earlier, different stimulation configurations and stimulus waveforms were implemented while exploring the optimal DBS parameters for therapeutic benefits. The STN population response for different types of DBS protocol was simulated. To study the neuroprotective effect, stimulation parameters which reduce the STN overactivity (Meissner et al., 2005) during dopamine depletion condition were chosen (Table 2). The biphasic stimulus pulse shows more therapeutic benefits than monophasic stimulus pulse; biphasic current alleviates the STN pathological activity without increasing the firing rate of STN population as a whole. The four-contact point type of stimulation configuration required lesser stimulus amplitude for producing the same effect when compared with the other two configurations. From these studies, we can say that four-contact point configuration with biphasic stimulus pulse gives maximum therapeutic benefits from the neuroprotective point of view.

**Table 2:** DBS parameter values obtained from the preliminary studies.

**Parameter(s)**	**SCP**	**FCP**	**MCP**
DBS frequency (*f*_*DBS*_) in Hz	130	130	130
Monophasic pulse width (δ_*DBS*_) in ms	100	100	100
Biphasic pulse width (δ_*DBS*_) in ms	200	200	200
Monophasic DBS amplitude (*A*_*DBS*_) in pA	650	650	650
Biphasic DBS amplitude (*A*_*DBS*_) in pA	1,000	1,000	1,000
Spread of the current (σ_*DBS*_)	5	2	0
Electrode contact point(s)	(16, 16)	(8, 8) (8, 24) (24, 8) (24, 24)	Many

*Hz, Hertz; ms, milliseconds; pA, picoamperes*.

To understand the neuroprotective therapeutic mechanism of DBS in PD (Benazzouz et al., 2000; Maesawa et al., 2004; Wallace et al., 2007; Spieles-Engemann et al., 2010; Musacchio et al., 2017), we have investigated some of the prominent hypotheses regarding the therapeutic effect of DBS viz., (1) excitation hypothesis, (2) inhibition hypothesis and most recent one (3) disruptive hypothesis (McIntyre et al., 2004; Chiken and Nambu, 2015).

The excitation hypothesis was implemented by direct stimulation of the STN population in the proposed excitotoxicity model. The simulation results show that DBS to STN diminishes the pathological synchronized activity but in turn increases the firing rate of the STN population which was not apt for neuroprotection. Next, we have implemented the inhibition hypothesis where antidromic activation of GPe neurons during STN-DBS is highlighted, thereby increasing the inhibitory drive to STN (Mandali and Chakravarthy, 2016). In this scenario also, the inhibitory drive from GPe was not sufficient to produce comprehensive neuroprotection (Figures 11, 12). On average FCP stimulus configuration produced better neuroprotective effect compared to other two configurations in both monophasic and biphasic current (Figures 11-2, 12-2). Moreover, MCP stimulus configuration results in worsening the disease progression by hastening the SNc cell loss in monophasic stimulus (Figure 11-3), but in biphasic stimulus, neuroprotection increased with higher levels of antidromic activation in all stages of therapeutic intervention (Figure 12-3).

Finally, the disruptive hypothesis was implemented by increasing the proportion of axonal and synaptic failures in STN population (Rosenbaum et al., 2014). From simulation results, it was observed that the progression of SNc cell loss was delayed or halted as the percentage of STN axonal and synaptic failures increased (Figures 11, 12). On average FCP stimulus configuration produced better neuroprotective effect compared to other two configurations in both monophasic and biphasic currents (Figures 11-2, 12-2). For the higher percentage of STN axonal and synaptic failures also, the neuroprotective effect was not pronounced in monophasic MCP DBS setting (Figure 11-3), but in biphasic MCP DBS setting neuroprotection increased with the higher percentage of STN axonal and synaptic failures (Figure 12-3).

## 4. Discussion

### 4.1. Excitotoxicity Model

The goal of this work was to develop a model which investigates the role of excitotoxicity in SNc cell loss, where excitotoxicity was caused by STN and precipitated by energy deficiency. The study suggests that excitotoxicity in SNc is initially driven by an energy deficit which leads to an initial dopamine reduction as a result of SNc cell loss. This initial dopamine reduction causes disinhibition of STN which in turns leads to excitotoxic damage due to excessive release of glutamate to its target nuclei including SNc (Rodriguez et al., 1998). The excitotoxicity which was driven by energy deficit, termed as “weak excitotoxicity,” results in increased vulnerability of SNc neurons to even physiological concentration of glutamate. The excitotoxicity which was driven by overactive excitatory STN neurons termed as “strong excitotoxicity” results in overactivation of glutamatergic receptors on SNc neurons (Albin and Greenamyre, 1992). In summary, it appears that the excitotoxic cause of SNc cell loss in PD might be initiated by weak excitotoxicity mediated by energy deficit, and followed by strong excitotoxicity, mediated by disinhibited STN.

The results from the proposed model reinforce the role of STN in regulating SNc cell loss (Hamani et al., 2004, 2017). The model results show that although cell loss was observed, there was no increased synchrony in the STN population which is a pathological marker of the PD condition (Lintas et al., 2012). Thus, the SNc cell loss and STN synchrony have a threshold-like relation where there is an increased STN synchrony only after substantial SNc cell loss. The initial SNc cell loss leads to further activation of STN by disinhibition, which in turn further activates SNc compensating for the dopamine loss, acting as a pre-symptomatic compensatory mechanism (Bezard et al., 2003). It was reported that the onset of PD symptoms occurs only after there is more than 50% SNc cell loss (Bezard et al., 2001). This was observed in our simulation results also where only at around 50–70% SNc cell loss there is in an increased STN synchrony. As a result of substantial SNc cell loss, decreased dopamine causes disinhibition of STN which in turn overactivates STN, eventually producing a runaway effect that causes an unstoppable SNc cell loss due to excitotoxic damage (Rodriguez et al., 1998). The threshold-like behavior of SNc cell loss and STN synchrony might also be facilitated by the inhibitory drive from GPe to STN: the proliferation of GPe-STN synapses (Fan et al., 2012) also acts as a presymptomatic compensatory mechanism. It was also reported that lesioning of GPe caused progressive SNc cell loss by increasing STN activity (Wright et al., 2002) and lesioning of STN proved to be neuroprotective (Wright and Arbuthnott, 2007).

To summarize, up to a point of stress threshold, SNc cells can survive indefinitely; but if, for any reason, there is loss of cells in SNc, and the SNc cell count falls below a threshold, from that point onwards, the aforementioned runaway effect kicks in leading to a progressive and irrevocable cell loss. Such cell loss is strongly reminiscent of cell loss due to neurodegeneration.

### 4.2. Neuroprotective Strategies

A variety of conventional therapies are simulated in the model to test their efficacy in slowing down or arresting SNc cell loss. Among the current therapeutics, glutamate inhibition, dopamine restoration, subthalamotomy and deep brain stimulation showed superior neuroprotective effects in the proposed model. Glutamate inhibition and subthalamotomy were successful in delaying or arresting the SNc cell loss by inhibiting the excitatory drive from STN to SNc (Lee et al., 2003; Wallace et al., 2007; Austin et al., 2010), and in case of dopamine restoration it is by replenishing the dopamine tone to the STN which in turn restores inhibition on itself (Olanow et al., 1998; Vaarmann et al., 2013), thereby diminishing STN-mediated excitotoxicity in SNc. The neuroprotective effect of glutamate inhibition, dopamine restoration and subthalamotomy was dependent on the dosage of glutamate inhibitors, the extent of dopamine restored and proportion of STN lesioned, respectively. As the disease progresses, the effect of glutamate inhibition on the rate of degeneration increased but in the case of dopamine restoration and subthalamotomy, it was decreased. In the late stages of disease progression, our computational study indicates that the neuroprotective effect of glutamate inhibition is more prominent than dopamine restoration and subthalamotomy.

From our study, it can be said that subthalamotomy mostly delays the SNc cell loss but very rarely halts it. This phenomenon was not much evident in the late stages of disease progression in the model which is consistent with the standard clinical understanding that the neuroprotective effect of subthalamotomy in advanced PD is not quite successful (Guridi and Obeso, 2015). Early treatment with subthalamotomy in PD can have a neuroprotective effect (Guridi et al., 2016) a trend that was reflected in our computational study. Another factor underlying the neuroprotective effect of subthalamotomy during the early stage of PD is the involvement of presymptomatic compensation mechanisms (Bezard et al., 2003). One of the compensatory mechanisms is the increased activity of STN before any significant striatal dopamine loss which leads to excess excitatory drive from STN to the remaining SNc cells to restore the dopamine loss due to initial cell loss (Bezard et al., 1999). This excess excitatory drive from STN eventually leads to excitotoxicity in SNc neurons. To overcome this excitotoxicity, subthalamotomy had to be applied very early after diagnosis of PD to have any neuroprotective effect (Guridi et al., 2016).

In our modeling study, we have explored various aspects of DBS protocol from stimulus waveforms to stimulus configurations and other DBS parameters. From the simulation results, it can be suggested that biphasic stimulus waveform with four-contact point stimulation configuration showed maximal neuroprotective effect since biphasic stimulus guarantees charge-balance in the stimulated neuronal tissue (Hofmann et al., 2011) and DBS parameters were given in the Table 2.

It has been reported that long-term stimulation (DBS) of STN results in the slowdown of the SNc cell loss in animal models (Maesawa et al., 2004; Temel et al., 2006; Wallace et al., 2007; Spieles-Engemann et al., 2010; Musacchio et al., 2017), but the mechanism behind the neuroprotective benefits of DBS is not elucidated. To understand the neuroprotective effect of DBS in PD, we have investigated three prominent hypotheses viz., excitation, inhibition and disruptive actions of DBS (Chiken and Nambu, 2015). In the excitation hypothesis, only DBS was applied which results in increased firing rate in STN and leads to more excitatory drive to SNc which eventually kills the SNc cells due to stress. Therefore, considering only the excitation hypothesis cannot explain the neuroprotective effect of DBS. Next, inhibition hypothesis was implemented where antidromic activation of GPe result in the increased inhibitory drive to STN (Mandali and Chakravarthy, 2016). In this scenario also, the neuroprotective effect of DBS could not be comprehensively explained. Finally, the disruptive hypothesis was implemented by increasing the axonal and synaptic failures in STN population during DBS therapy (Rosenbaum et al., 2014). From simulation results, it was observed that the progression of SNc cell loss kept on delaying as the percentage of STN axonal and synaptic failure increased. Therefore, it can be inferred that DBS blocks the propagation of pathological oscillations occurring in STN to other nuclei; in other words DBS disrupts the information transfer through the stimulation site, producing neuroprotection effect in SNc (Ledonne et al., 2012).

### 4.3. Limitations and Future Directions

Though the model captures the exciting results of excitotoxicity, it is not without limitations. The timescales which are represented in the results of the proposed model are not realistic, as the neurodegeneration which occurs over the years in PD was exhibited in a few tens of seconds in the model. This limitation is inevitable due to the practical challenges faced by computer simulations since it is impractical to simulate the model for months and years. The difficulty arises due to the fact that the simulation must span widely separated time scales - sub-millisecond time scales to describe spiking activity and years to describe neurodegenerative processes.

The major inputs to the SNc neurons come from the striatum which was not included in the model. As our objective was to investigate the extent of STN-mediated excitotoxicity in SNc, we avoided any other structures which can influence this phenomenon at present.

In the proposed model, the variability of stress threshold, which is analogous to an apoptotic threshold (that can be broadly associated with the available energy represented as (ATP/ADP) ratio), is sensitive enough to alter the model results is a constant parameter. In order to achieve variability in this parameter, astrocytic and vascular network-level models can be introduced. With the astrocyte layer introduced, the effect of astrocytes on the therapeutic effect of DBS can be explored (Fenoy et al., 2014).

In the future, we plan to simulate the SNc activity using a detailed biophysical model to study the dynamics at the molecular level and also to investigate the cellular pathways related to PD pathology. We would like to include Spike-timing-dependent plasticity (STDP) learning in STN population for the long-term effect of DBS (Ebert et al., 2014).

Our hypothesis behind this whole study is to understand the pathogenesis of PD as cellular energy deficiency in SNc as a cause. As Wellstead and Cloutier pointed out (Wellstead and Cloutier, 2011), PD should be understood by placing the failure of brain energy delivery mechanisms in the center as a core pathological process, with other manifestations of pathology as deriving from that core process (see the Figure 12 in Wellstead, 2010).

## Code Accessibility

The code of the proposed excitotoxicity model is available on ModelDB server (McDougal et al., 2017), and accesscode will be provided on request. (https://senselab.med.yale.edu/modeldb/enterCode.cshtml?model=244384).

## Author Contributions

VM and VC: conceived, developed the model and prepared the manuscript. AM: conceived and developed the model. SR: prepared the manuscript.

### Conflict of Interest Statement

The authors declare that the research was conducted in the absence of any commercial or financial relationships that could be construed as a potential conflict of interest.
